# Smoking-associated Downregulation of FILIP1L Enhances Lung Adenocarcinoma Progression Through Mucin Production, Inflammation, and Fibrosis

**DOI:** 10.1158/2767-9764.CRC-22-0233

**Published:** 2022-10-18

**Authors:** Mijung Kwon, Genesaret Rubio, Haitao Wang, Gregory Riedlinger, Asha Adem, Hua Zhong, Daniel Slegowski, Louisa Post-Zwicker, Anshruta Chidananda, David S. Schrump, Sharon R. Pine, Steven K. Libutti

**Affiliations:** 1 Rutgers Cancer Institute of New Jersey, New Brunswick, New Jersey.; 2 Thoracic Surgery Branch, Center for Cancer Research, NCI, Bethesda, Maryland.; 3 Department of Pathology, Robert Wood Johnson Medical School, Rutgers University, New Brunswick, New Jersey.; 4 Departments of Pharmacology and Medicine, Robert Wood Johnson Medical School, Rutgers University, New Brunswick, New Jersey.

## Abstract

Lung adenocarcinoma (LUAD) is the major subtype in lung cancer, and cigarette smoking is essentially linked to its pathogenesis. We show that downregulation of Filamin A interacting protein 1-like (FILIP1L) is a driver of LUAD progression. Cigarette smoking causes its downregulation by promoter methylation in LUAD. Loss of FILIP1L increases xenograft growth, and, in lung-specific knockout mice, induces lung adenoma formation and mucin secretion. In syngeneic allograft tumors, reduction of FILIP1L and subsequent increase in its binding partner, prefoldin 1 (PFDN1) increases mucin secretion, proliferation, inflammation, and fibrosis. Importantly, from the RNA-sequencing analysis of these tumors, reduction of FILIP1L is associated with upregulated Wnt/β-catenin signaling, which has been implicated in proliferation of cancer cells as well as inflammation and fibrosis within the tumor microenvironment. Overall, these findings suggest that down-regulation of FILIP1L is clinically relevant in LUAD, and warrant further efforts to evaluate pharmacologic regimens that either directly or indirectly restore FILIP1L-mediated gene regulation for the treatment of these neoplasms.

**Significance:**

This study identifies FILIP1L as a tumor suppressor in LUADs and demonstrates that downregulation of FILIP1L is a clinically relevant event in the pathogenesis and clinical course of these neoplasms.

## Introduction

In the United States, lung cancer is the leading cause of cancer-related mortality in men and women, and presently accounts for nearly 25% of all cancer deaths (1). An estimated 236,740 lung cancers will be diagnosed and 130,180 deaths will be attributed to lung cancer during 2022 (1). The 5-year relative survival rate for lung cancer is 22%, which is much lower than that of many other cancers (1). Approximately 81% of lung cancer deaths in 2022 will be directly related to cigarette smoking, with an additional 3% due to second-hand smoke (1, 2). Tobacco carcinogens induce characteristic genomic as well as epigenomic alterations including copy-number variations and point mutations as well as aberrant DNA methylation that collectively induce genomic instability and malignant transformation in airway epithelial cells (3–9). The fact that cigarette smoke induces time- and dose-dependent chromatin alterations in human respiratory epithelial cells (10, 11), which sensitize these cells to transformation by a single oncogenic event (e.g., *KRAS* mutations; ref. 12) attest to the significance of epigenetic perturbations during pulmonary carcinogenesis.

Recent transcriptomic and epigenomic analyses have demonstrated that *Filamin A interacting protein 1-like* (*FILIP1L*) is a key gene that is repressed by promoter DNA methylation in normal human small airway and bronchial epithelial cells following exposure to cigarette as well as hookah tobacco smoke (13). These findings extend our previous observations that *FILIP1L* is a novel tumor suppressor gene that is repressed by promoter methylation in several cancer histologies (14–18). Downregulation of FILIP1L is associated with chemoresistance and poor prognosis in ovarian and colon cancer (19, 20). Mechanistically, FILIP1L promotes β-catenin degradation and suppresses epithelial-to-mesenchymal transition (EMT), thereby inhibiting metastases and chemoresistance in ovarian cancer (15, 19). In addition, FILIP1L regulates the degradation of PFDN1 (18), a molecular chaperone which activates Wnt/β-catenin signaling-mediated EMT, thereby facilitating cell migration, invasion, and metastasis in cancer cells (21); overexpression of PFDN1 is associated with poor prognosis in colon cancer and non–small cell lung cancer (NSCLC; refs. 22, 23). Collectively, these observations suggest that epigenetic repression of *FILIP1L* enhances the malignant phenotype of cancer cells via Wnt/β-catenin signaling.

Mucus functions to prevent water loss and facilitates removal of inhaled foreign substances such as particulate matter and microbes, thereby maintaining normal pulmonary physiology. Secreted mucin proteins such as MUC5AC and MUC5B are major macromolecular components of airway mucus and play important roles in regulation of innate immune function in the lungs (24). Cell surface–associated mucins such as MUC1, MUC4, MUC16, and MUC20 attach to airway epithelial microvilli and cilia to establish an osmotic barrier (24). Coordinated interactions of secreted and membrane-associated mucins in healthy airways are critical for normal mucociliary clearance. Airway mucin hypersecretion and/or overexpression has been linked to chronic inflammatory lung diseases such as cystic fibrosis, asthma, idiopathic pulmonary fibrosis, and chronic obstructive pulmonary disease (24), some of which are associated with increased lung cancer risk (25). In addition, mucins increase growth and survival of lung cancer cells (26), and mucin hypersecretion and/or overexpression is significantly associated with enhanced metastasis and poor prognosis in NSCLC (27–29).

Recently, we reported that FILIP1L downregulation increases mucus production and enhances the malignant phenotype of colon carcinoma cells (18). The current study was undertaken to examine the mechanisms and potential implications of *FILIP1L* repression in NSCLC. Findings presented herein suggest that downregulation of FILIP1L is a clinically relevant event in the pathogenesis and clinical course of lung adenocarcinomas (LUAD).

## Materials and Methods

### Bioinformatic Analysis

LUAD, lung squamous carcinomas (LUSC), and other pan-cancer cohort datasets were derived from the publicly available The Cancer Genome Atlas (TCGA) databases and the Genotype-Tissue Expression (GTEx) projects. mRNA expression and DNA methylation data as well as clinical information were downloaded from UCSC Xena (https://xena.ucsc.edu/, RRID:SCR_018938). For most datasets, the clinical characteristics, including histologic type, grade, stage, age, smoking status, and survival data, were available. We obtained 31 mRNA RNA-Seq-HTSeq-fragments per kilobase of exon model per million mapped reads (FPKM) data from TCGA databases to analyze unpaired tumor versus normal tissues as well as paired tumor versus its nontumor adjacent tissue (NAT) samples. TCGA methylation data were generated by the Illumina Human Methylation 450K BeadChip. We assigned DNA methylation values for the *FILIP1L* gene with the average β value of the probes mapped to the promoter region, including TSS200 (region from –200 bp upstream to the transcription start site), 1stExon (the first exon), TSS1500 (from −200 to −1,500 bp upstream of transcription start site) and 5′ untranslated region in order.

Samples from patients with cigarette exposures greater or lower than the median (extensive or limited exposures, respectively) were determined as follows. **Cigarettes_per_day** The average number of cigarettes smoked per day (https://docs.gdc.cancer.gov/Data_Dictionary/viewer/#?view=table-definition-view&id=exposure&anchor=cigarettes_per_day) for patients with LUAD and LUSC were determined as 1.26 and 2.19, respectively. Following linear regression transformation based on fitting linear models, **pack_years_smoked** (numeric computed value to represent lifetime tobacco exposure defined as a number of cigarettes smoked per day × number of years smoked divided by 20) for patients with LUAD and LUSC were determined as 23 and 40, respectively.

### Cell Culture and Development of Stable Clones

The following cell lines were purchased from ATCC: human lung cancer lines including H1573 (CRL-5877, RRID:CVCL_1478), H1299 (CRL-5803, RRID:CVCL_0060), H322 (Discontinued, RRID:CVCL_1556), H1792 (CRL-5895, RRID: CVCL_1495), H2087 (CRL-5922, RRID:CVCL_1524), H2030 (CRL-5914, RRID:CVCL_1517), H1693 (CRL-5887, RRID:CVCL_1492), H1944 (CRL-5907, RRID:CVCL_1508), and H441 (CRM-HTB-174, RRID:CVCL_1561). mTC11 mouse cell line was kindly provided by Dr. Bergo (Karolinska Institutet; ref. 30). Cells were cultured following the manufacturer's guidelines, and passaged up to five times after each thawing. All cell lines were authenticated using short tandem repeat profiling, and were routinely tested for *Mycoplasma* contamination using Universal *Mycoplasma* Detection Kit (ATCC, 30-1012K).

FILIP1L-knockdown clones were generated from H1944 and H441 cells. Cells were transduced by lentiviruses purchased from Applied Biological Materials. Lentiviruses encoding either scrambled short hairpin RNA (shRNA) or *FILIP1L*-shRNA were used. Pooled lentiviruses from four different sequences of *FILIP1L*-shRNA were used as described previously (18). To generate *Filip1l*-knockdown clones from mTC11 mouse cells**,** lentiviruses from two different sequences of *Filip1l*-shRNA were used (Target a: CTTCAGTCACTGGAAGCAATTGAGAAAGA and Target c: AGAGCCTCATTCCTCTGGAAAGAGCAGTG). Following transduction, resistant cells were screened by puromycin selection and mixed clones were selected by immunoblot.

### Mouse Xenograft Model

All use of vertebrate animals described in this study was conducted in accordance with NIH regulations and was approved by the Animal Use Committee of Rutgers University (New Brunswick, NJ). Indicated number of lung cancer clones were suspended in growth factor–reduced Matrigel [Corning #356231; 1:1 ratio (v:v)] and subcutaneously injected in 8-week-old female nude mice (Taconic, catalog no. TAC:nmrinu, RRID:IMSR_TAC:nmrinu). Tumor growth was measured for indicated times, and tumor weights were measured after sacrifice. Xenograft tumors were fixed in 10% neutral buffered formalin and subject to IHC analysis.

### Mouse Syngeneic Allograft Model

Indicated number of mTC11 clones were subcutaneously injected in 8-week-old male C57BL6/J mice (IMSR catalog no. JAX_000664, RRID:IMSR_JAX:000664). The same following procedures were performed as described in the previous section.

### *Filip1l* Conditional Knockout Mice

*Filip1l*-floxed mice were generated as described previously (18). *Filip1l^fl/fl^* mice were subsequently generated and were crossed with *Ubc-CreER^T2^* transgenic mice (Jackson laboratories #007001, RRID:IMSR_JAX:007001) to generate inducible systemic *Filip1l^fl/fl^; Ubc-CreER^T2^* knockout mice. To induce Cre recombinase-mediated knockout of *Filip1l* gene, tamoxifen (TAM; 160 mg/kg/day) was injected intraperitoneally for 5 consecutive days. Lungs were fixed in 10% neutral buffered formalin and subject to IHC analysis. Three 10-μm-thick sections were cut from each formalin-fixed paraffin-embedded (FFPE)-lung tissue block, and genomic DNAs and total RNAs were purified using AllPrep DNA/RNA FFPE Kit (Qiagen #80234). The combined *Filip1l* allele was detected using primers, ACATGCGTAATGGCTCAAGCAAGC and GGAGAATGTCCAGAAGTTTATGTC. The housekeeping gene, *m18S RNA* was detected using primers, CTTAGAGGGACAAGTGGCG and ACGCTGAGCCAGTCAGTGTA.

### Lentiviral Delivery of Cre Recombinase by Intratracheal Infection

Lentiviruses were purchased from Viral Vector Core Laboratories of University Iowa (#FIVCMVCre VSVG). Diluted lentiviruses (5 × 10^5^ TU per mouse) were delivered by intratracheal inhalation into 8–10 weeks old C57BL6/J and *Filip1l^fl/fl^* mice as described previously (31).

### Clinical Specimens

FFPE tissue blocks of deidentified human samples of normal lung and LUAD were obtained from Biorepository Services at the Rutgers Cancer Institute of New Jersey, under our Institutional Review Board exemption. Tissue microarray of human LUAD was constructed previously (32). IHC staining was carried out and a clinical pathologist scored the staining under blinded conditions. FILIP1L cytoplasmic staining was scored according to the staining intensity [categorized as 0 (absent), 1 (weak), 2 (moderate), or 3 (strong)] as well as the percentage of staining [0%–100%]. The final expression score was calculated by multiplying the intensity and the percentage of staining resulting in a score of 0 to 300.

### Time-lapse Imaging

Mitotic length and time to cytokinesis completion were measured by live imaging of H1944 and H441 clones as described previously (18). Slight modifications in the experimental procedures were as follows. Cells were incubated with SPY-595 DNA and SPY-650-tubulin fluorescent dyes (Cytoskeleton) for 30 minutes. Images acquired over the initial 6 hours were used to quantify data.

### qRT-PCR

Total RNA preparation and qRT-PCR were performed as described previously (14). The gene-specific primers used with SYBR Green reagent are written in Supplementary Data.

### Immunoblot

Experimental details for immunoblotting were followed as described previously (15). Densitometric analysis was performed using ImageJ (RRID:SCR_003070) on scanned images of immunoblots as described previously (18). Antibody list used in assays such as immunoblot, IHC, and immunofluorescence is shown in Supplementary Data.

### IHC

Experimental details were followed as described previously (19). Images were acquired by AxioImager microscope (Zeiss). For stitched images, they were acquired by EVOS FL Auto microscope (Thermo Fisher Scientific). To detect mucin proteins, sections were stained with 1% Alcian Blue, pH 2.5 followed by periodic acid–Schiff (PAS) reagent. For the quantification of mucin proteins, sections were stained with PAS only so that quantification can be done on one color staining of PAS-positive area in a straightforward manner. Detailed quantification procedures for PAS and Picro-Sirius Red stainings were written in Supplementary Data.

### Immunofluorescence Staining and Quantification

Experimental details were followed as described previously (15). Images were acquired by an AxioCam HRM camera (Yokogawa) at 20 × objective magnification (*z* stack of 3 μmol/L thickness) on a Spinning disc confocal microscope (Zeiss; Observer Z1). Acquired images were then analyzed for β-catenin intensity by software such as ZEN (Zeiss, RRID:SCR_013672) and Cell Profiler (RRID:SCR_007358; ref. 33). Detailed quantification procedures for β–catenin intensity were written in Supplementary Data. Quantification procedures for Ki67 area were followed as described previously (18).

### Immunoprofiling

Fresh syngeneic allograft tumors and spleens were procured from each mouse (5 mice per group). The weights of tumors and spleens were measured and used for normalizing cell counts. Tumors were dissociated using mouse tumor dissociation kit (Miltenyi Biotec, #130096730)/gentleMACS Octo Dissociator with heaters with hard tumor protocol. Dissociated tissues were filtered with 70 μm Miltenyi Smart Strainer (#130110916), followed by red blood cell lysis (Sigma RBC lysing buffer, Hybri Max, #R7757) and debris removal. Live cell counts were calculated by NucGreen Dead 488 staining (Thermo Fisher Scientific, #R37109), and 1 × 10^7^ cells per sample were used for the staining with various immune cell markers. Markers such as myeloid cell immunophenotyping (dendritic cells, neutrophils, monocytes, and macrophages), T-cell function (naïve, effector, memory, extravasation, activation and exhaustion markers) and intracellular markers [regulatory T cells (Treg) and proliferating cells] were used. Flow cytometry analysis of multiplexed cells was performed on a Cytek Aurora 5-laser cytometer using the SpectroFlow software package version 2.2 (Cytek Biosciences; 4 L 16 UV 16V 14B 8R). FACS analysis was further performed on the exported fcs files using FlowJo software (RRID:SCR_008520).

### RNA Sequencing

Total RNAs were prepared from snap-frozen mouse syngeneic allograft tumors using RNeasy Plus Mini Kit (Qiagen #74134). Sample quality control (QC), library preparations, sequencing reactions, and bioinformatic analyses were conducted at GENEWIZ/Azenta Inc. according to their standard protocol, which is summarized as follows: Integrity of RNA samples was checked using TapeStation (Agilent Technologies, RRID:SCR_019547). RNA-seq libraries were prepared using the NEBNext Ultra II RNA Library Prep Kit for Illumina (New England Biolabs). mRNAs were initially enriched with Oligod(T) beads. Sequencing libraries were validated on the Agilent TapeStation, and quantified by using Qubit 2.0 Fluorometer (Thermo Fisher Scientific) as well as by qPCR. Sequencing libraries were multiplexed and clustered onto a flowcell. Samples were sequenced using a 2 × 150 bp paired end configuration in the Illumina HiSeq instrument. Image analysis and base calling were conducted by the HiSeq Control Software (HCS). Raw sequence data (.bcl files) were converted into fastq files and demultiplexed using Illumina bcl2fastq 2.20 software. One mismatch was allowed for index sequence identification. Sequence reads were trimmed to remove adapter sequences using Trimmomatic v.0.36 (RRID:SCR_011848), and the trimmed reads were mapped to the Mus musculus reference genome available on ENSEMBL using the STAR aligner v.2.5.2b (RRID:SCR_004463). Unique gene hit counts were calculated by using feature Counts from the Subread package v.1.5.2., and were used for downstream differential expression analysis. Using DESeq2, a comparison of gene expression between control and *Filip1l*-knockdown groups was performed. The Wald test was used to generate *P* values and log_2_ fold changes. Genes with adjusted *P* values <0.05 and absolute log_2_ fold changes >1 were called as differentially expressed genes for each comparison. Gene ontology analysis was performed on the statistically significant set of genes by implementing the software GeneSCF v.1.1-p2. The goa_MusMusculus gene ontology (GO) list was used to cluster the set of genes based on their biological process and determine their statistical significance. principal component analysis was performed using the "plotPCA" function within the DESeq2 R package (RRID:SCR_000154). The plot shows the samples in a two-dimensional plane spanned by their first two principal components. The top 500 genes, selected by highest row variance, were used to generate the plot.

### Statistical Analysis

All statistical analyses for bioinformatics data were performed using the R program (version 4.0.0). Unpaired two-sided *t* test was applied to determine the differential mRNA expression between the tumor and normal tissues. For the comparison between the tumor and its NATs, paired *t* test was used. Pearson correlation coefficient was achieved to determine the correlation between mRNA expression, methylation and cigarette exposures. Bar graphs are presented as the mean ± SEM. Statistical analyses other than bioinformatic analyses were performed using an unpaired, two-tailed Student *t* test [GraphPad Prism 6.0 (RRID:SCR_002798)]. Differences were considered statistically significant at *P* < 0.05.

### Data Availability Statement

The data generated in this study are publicly available in Gene Expression Omnibus (RRID:SCR_005012) at GSE208080.

## Results

### FILIP1L Downregulation is Associated with Smoking History in Patients with LUAD

**FIGURE 1:**
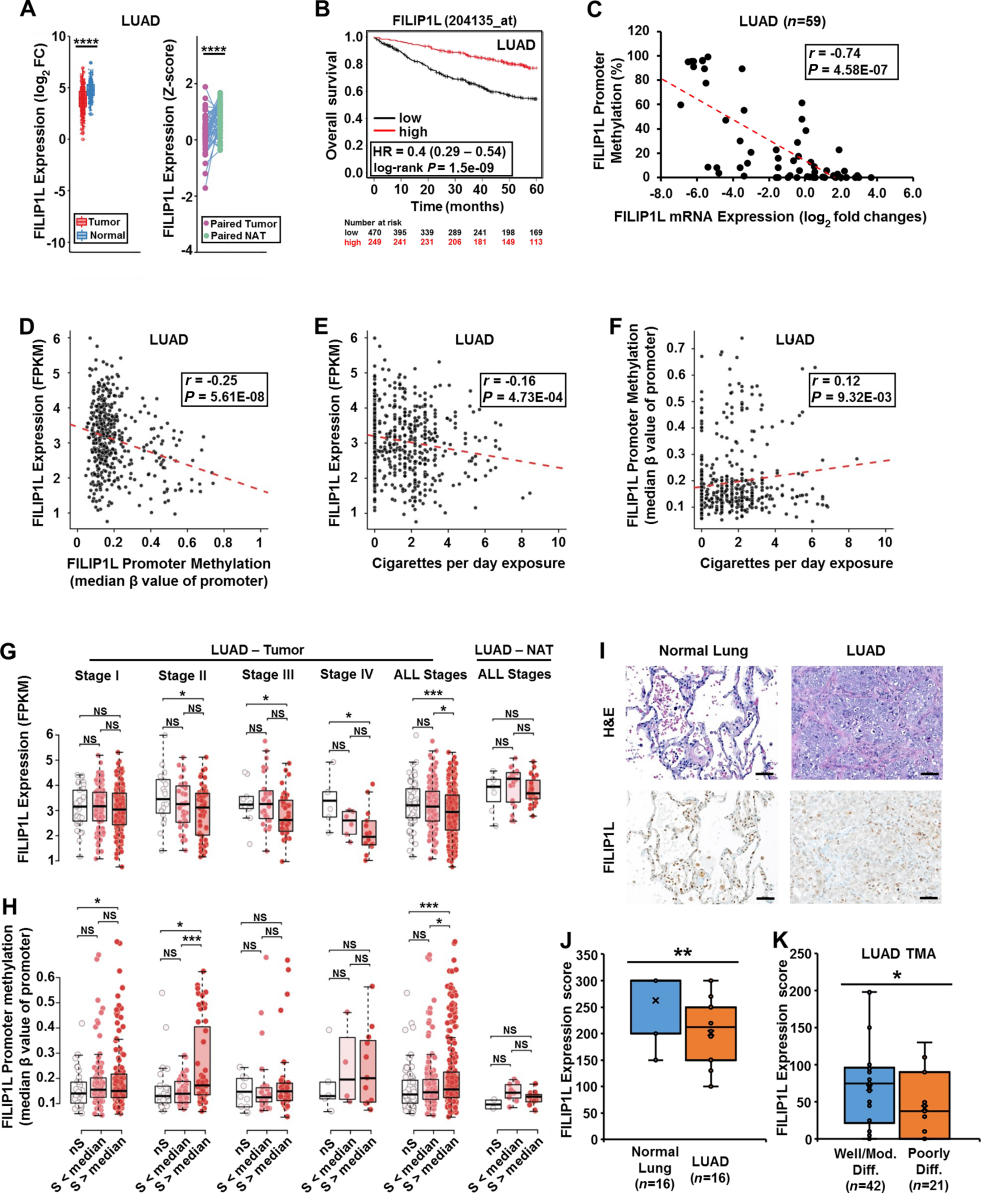
FILIP1L downregulation is associated with smoking history in patients with LUAD. **A,***FILIP1L* mRNA expression between tumor and normal tissues from patients with LUAD were compared. Plots shown are comparison between tumors and normal lungs (left) or between paired tumors and NATs (right). **B,** OS of patients with LUAD whose *FILIP1L* expression was either low or high was analyzed in Kaplan–Meier plots. Data are derived from Kaplan–Meier Plotter public databases (https://kmplot.com/analysis/index.php?p=service). Affymetrix ID used was 204135_at. Cut-off values of 1,718 were determined by auto select best cut-off option. Using the selected parameters, the analysis was run on 719 patients. **C,** Relationship between *FILIP1L* mRNA expression (*x* axis) and *FILIP1L* promoter methylation (*y* axis) is shown in cell lines derived from LUAD (*P* values shown are by Spearman rank correlation method). The *x* axis represents *FILIP1L* mRNA expression of log_2_ fold changes from RNA-seq data. The *y* axis represents percent methylation of the average overall methylation for all available CG sites in *FILIP1L* promoter. Data were derived from Cancer Cell Line Encyclopedia public databases (https://sites.broadinstitute.org/ccle/). LUAD samples from TCGA databases were analyzed for the relationship between *FILIP1L* promoter methylation (*x* axis; median β value of *FILIP1L* promoter methylation) and *FILIP1L* mRNA expression (*y* axis; FPKM values from RNA-seq data; **D**); the daily amount of cigarette exposures (*x* axis) and *FILIP1L* mRNA expression (*y* axis; **E**); the daily amount of cigarette exposures (*x* axis) and *FILIP1L* promoter methylation (*y* axis; **F**). *FILIP1L* mRNA expression **(G)** or *FILIP1L* promoter methylation **(H)** was compared between nonsmokers (nS), smokers exposed less than the median of tobacco dose (S < median) and smokers exposed more than the median of tobacco dose (S > median) in the stage I–IV tumors of patients with LUAD. Comparison in the samples with all stages and NAT are also shown. **I,** Representative images of H&E staining and FILIP1L IHC staining in non-tumor adjacent lung tissues (NATs, *n* = 16) and LUAD (*n* = 16) are shown. Scale bar = 50 μm. **J,** FILIP1L expression in immunohistochemically stained slides (as shown in **I**) was compared between matched normal and LUAD samples. Expression score was carried out as described in Materials and Methods. **K,** FILIP1L expression in immunohistochemically stained tissue microarray consisted with LUAD tumors was compared between well/moderately and poorly differentiated samples. *, **, ***, and **** indicate *P* < 0.05, *P* < 0.01, *P* < 0.001 and *P* < 0.0001, respectively.

We have shown that *FILIP1L* mRNA levels are lower in LUAD and LUSC compared with unpaired normal lung tissues (13). To further examine this issue, we evaluated *FILIP1L* mRNA expression in more extensive NSCLC databases including paired tumor and NAT samples. Consistent with our previous report (13), *FILIP1L* mRNA expression was significantly decreased in both LUAD (Fig. 1A) and LUSC (Supplementary Fig. S1A) tumors compared with normal lung tissues. We next examined whether *FILIP1L* expression was associated with prognosis in patients with NSCLC. Low intratumoral *FILIP1L* mRNA expression correlated with decreased overall survival (OS) in patients with LUAD (Fig. 1B) as well as LUSC (Supplementary Fig. S1B). Additional analysis of unpaired and paired samples in TCGA and GTEx databases demonstrated that *FILIP1L* mRNA was significantly downregulated in many other cancer types compared with respective normal tissues (Supplementary Fig. S1C and S1D), suggesting that repression of this tumor suppressor gene is a common event in the pathogenesis of human malignancies.

Our previous studies have suggested that FILIP1L is downregulated by promoter methylation in various cancer histologies (14, 16). As such, we used a public database to more comprehensively examine the association of *FILIP1L* mRNA expression and *FILIP1L* promoter DNA methylation in a panel of lung cancer lines. As shown in Fig. 1C, a significant, negative correlation was observed between *FILIP1L* mRNA expression and *FILIP1L* promoter methylation in LUAD cell lines; this inverse relationship was not observed in LUSC cell lines (Supplementary Fig. S1E).

We next examined whether *FILIP1L* mRNA expression and *FILIP1L* promoter methylation were associated with smoking history in patients with NSCLC. Analysis of TCGA datasets demonstrated significant inverse correlations between *FILIP1L* mRNA expression and *FILIP1L* promoter methylation in both LUAD (Fig. 1D) and LUSC (Supplementary Fig. S1F) samples. Significant inverse or positive associations were observed between *FILIP1L* mRNA expression or *FILIP1L* promoter methylation, respectively and extent of cigarette exposure in LUAD samples (Fig. 1E and F); these associations were not evident for LUSC (Supplementary Fig. S1G and S1H).

Next, we examined whether *FILIP1L* expression and *FILIP1L* promoter methylation were related to tumor progression. A clear downward trend of *FILIP1L* mRNA levels was observed in stages II, III, and IV LUAD when comparing smokers with nonsmokers; these differences within the aforementioned stages were significant when comparing samples from patients with cigarette exposures greater than the median (extensive exposures; defined in Materials and Methods) to nonsmokers (Fig. 1G); although *FILIP1L* mRNA levels in LUAD from smokers with heavy exposures were lower than those detected in LUAD from individuals with exposures less than the median (limited exposures) for stages II–IV, these differences were not significant when comparing samples within each respective stage. However, these differences were significant following combined analysis of all stages. Overall, these findings suggest a dose-dependent effect of cigarette smoking on *FILIP1L* expression in LUAD. *FILIP1L* promoter methylation was more pronounced in individuals with extensive cigarette smoke exposures in stage I and stage II LUAD, but was not evident for more advanced stages (Fig. 1H). However, analysis of all stages demonstrated significantly higher DNA methylation levels within the *FILIP1L* promoter in LUAD samples from individuals with extensive smoke exposures relative to smokers with lower exposures or non-smokers. No consistent differences were observed between *FILIP1L* mRNA levels and *FILIP1L* promoter methylation status relative to smoking histories in LUSC (Supplementary Fig. S1I and S1J).

We next examined stage-related changes in *FILIP1L* mRNA and *FILIP1L* promoter methylation levels in LUAD from nonsmokers, and smokers with exposures less than or more than the median. No significant stage-related changes in either *FILIP1L* mRNA or promoter DNA methylation levels were evident in nonsmokers (Supplementary Fig. S1K and S1L). In LUAD samples from patients with cigarette exposures less than the median, significant changes in both mRNA and promoter methylation levels were evident only in stage IV tumors. In samples from individuals with cigarette exposures above the median, *FILIP1L* mRNA levels were decreased in stage IV LUAD relative to stages I, II, or III tumors. DNA methylaton levels were significantly higher in stage IV tumors in smokers with exposures below as well as above the median. Once again, when comparing *FILIP1L* mRNA levels and *FILIP1L* promoter methylation levels in all LUAD samples, clear differences were evident in *FILIP1L* mRNA and DNA methylation levels in individuals with extensive cigarette exposures compared with those with limited exposures or non-smokers. No significant differences were evident when comparing *FILIP1L* mRNA and *FILIP1L* promoter methylation levels in normal lung tissues adjacent to LUAD (all stages) relative to patient smoking status (Fig. 1G and H). Collectively, these findings suggest that *FILIP1L* downregulation and *FILIP1L* promoter DNA methylation are associated with locally advanced or metastatic LUAD, particularly in patients with extensive cigarette exposures.

Having demonstrated decreased *FILIP1L* mRNA expression in LUAD, we examined FILIP1L protein levels in these patient samples. IHC staining revealed that FILIP1L localizes in alveolar pneumocytes of the normal lung, and its expression is reduced in LUAD samples (Fig. 1I; representative). FILIP1L expression was significantly decreased in LUAD compared with their matched NATs (Fig. 1J); significantly less FILIP1L protein expression was detected in poorly differentiated compared with well/moderately differentiated LUAD (Fig. 1K). Overall, these results suggest that FILIP1L is downregulated (at least in part) by promoter methylation, which is associated with heavy smoking in patients with LUAD, and that FILIP1L downregulation is associated with a more aggressive (less differentiated) tumor histology.

### FILIP1L Knockdown Induces Cytokinesis Defects in Lung Cancer Cells *In Vitro* and Enhances Their Growth *In Vivo*

**FIGURE 2:**
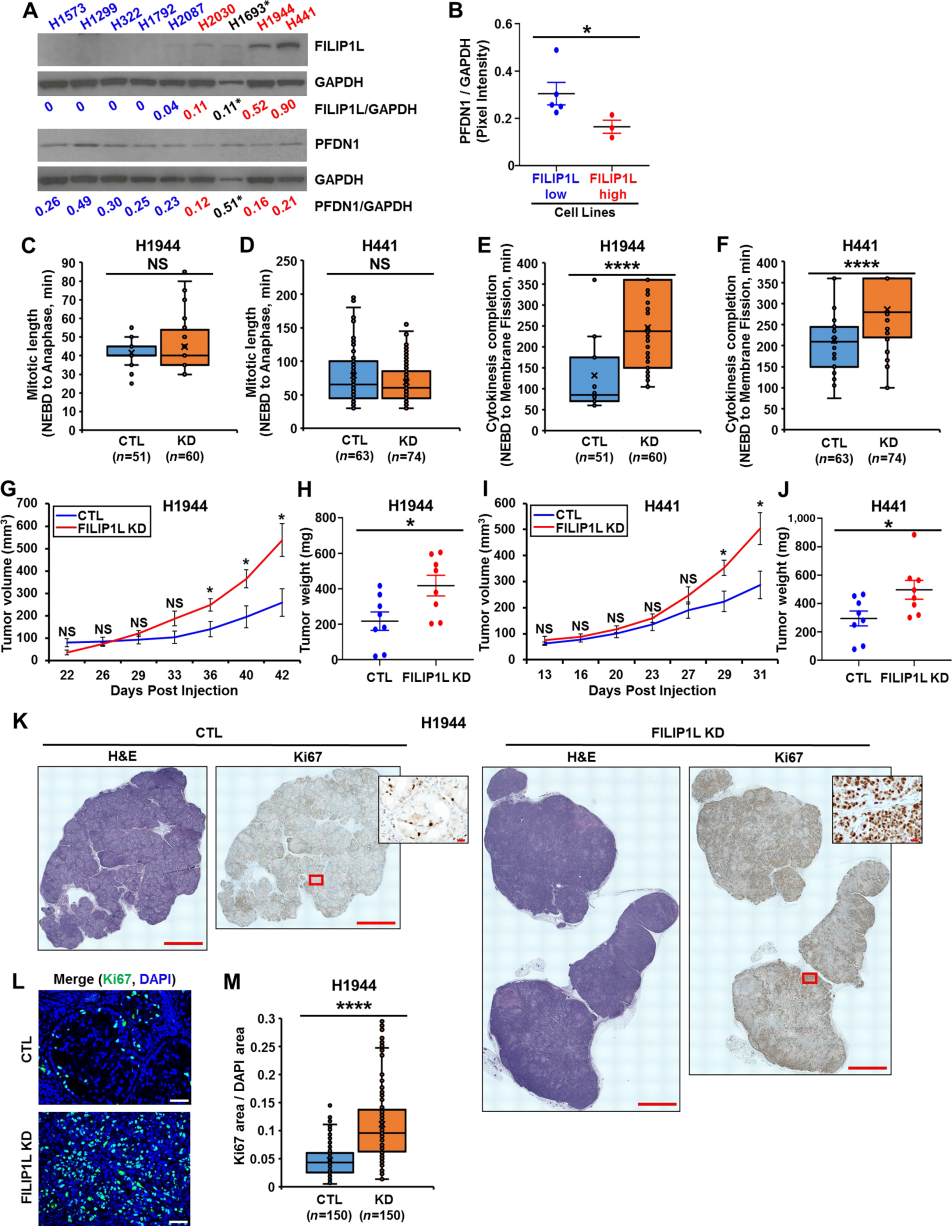
FILIP1L knockdown induces cytokinesis defects in lung cancer cells *in vitro* and enhances their growth *in vivo*. **A,** Protein levels of FILIP1L and PFDN1 were determined by immunoblotting in various lung cancer cell lines. GAPDH control is also shown. Note H1693 cells express a considerably less GAPDH. By densitometric quantification, the amount of FILIP1L, PFDN1, and GAPDH was determined. The ratio of FILIP1L/GAPDH and PFDN1/GAPDH is shown. **B,** On the basis of FILIP1L/GAPDH ratio, cell lines were divided into FILIP1L-low (H1573, H1299, H322, H1792, and H2087) and FILIP1L-high groups (H2030, H1944, and H441), and plotted against PFDN1/GAPDH ratio. H1693 cells were excluded from the comparison due to the off the scale resulting from considerably less GAPDH. **C–F,** Time-lapse imaging of H1944 and H441 clones that were incubated with SPY-595 DNA and SPY-650-tubulin fluorescent dyes. Mitotic length (**C** for H1944; **D** for H441) and cytokinesis completion (**E** for H1944; **F** for H441) from control and FILIP1L knockdown clones were quantified from three independent experiments. **G–M,** H1944 and H441 clones (4 × 10^6^) of either control or FILIP1L-knockdown derivatives were subcutaneously injected into the Nude mice (8 mice per cell line). Tumor growth (**G** for H1944; **I** for H441) was measured, every 2–3 days for a total of 42 and 31 days for H1944 and H441, respectively. The *y* axis represents tumor volume that was calculated by the formula: (length × width × height × 0.52). Tumor weights at the time of sacrifice were measured (**H** for H1944; **J** for H441). **K,** H1944 xenograft tumors from either control or FILIP1L-knockdown derivatives were fixed, and stained with H&E. They were also immunohistochemically stained for Ki67. Stitched images are shown. Scale bar = 2,000 μm. Magnified images of Ki67 staining (Scale bar = 20 μm) from the boxed areas of tumor regions are shown in insets. **L** and **M,** H1944 xenograft tumors were immunofluorescently stained for Ki67 (green). Nuclei were counterstained with DAPI (blue), and merged fluorescent images are shown **(L)**. Scale bar = 50 μm. Ki67-positive areas from the immunofluorescence staining were quantified **(M).** Fifty random fields per mouse were quantified (three mice each). * and **** indicate *P* < 0.05 and *P* < 0.0001, respectively.

We have recently shown that FILIP1L regulates proteasome-dependent degradation of the molecular chaperone PFDN1, and that increased PFDN1 expression, resulting from downregulation of FILIP1L leads to cytokinesis defects and enhanced tumor growth in colon cancer (18). Thus, we examined the relationships between FILIP1L and PFDN1 expression in the context of lung cancer. Preliminary immunoblot experiments (Fig. 2A and B) demonstrated that FILIP1L expression coincided inversely with PFDN1 protein levels in lung cancer cell lines.

We first asked whether FILIP1L knockdown leads to cytokinesis defects in lung cancer cells. Using lentiviral transduction, we knocked down FILIP1L in H1944 and H441 LUAD lines; immunoblotting confirmed that knockdown of FILIP1L resulted in increased PFDN1 expression (Supplementary Fig. S2A). FILIP1L knockdown as well as control (CTL) clones from H1944 and H441 cell lines were marked for DNA and tubulin, and cells entering mitosis were monitored every 5 minutes using live imaging techniques. In line with our previous observations pertaining to colon cancer cells, mitotic length [time between nuclear envelope breakdown and anaphase (34–36)] was not significantly different between FILIP1L-knockdown and control clones in either cell line (Fig. 2C and D). However, the time for membrane fission, the final step in cytokinesis (37, 38), was significantly delayed in FILIP1L-knockdown clones compared with controls (Fig. 2E and F). We next asked whether FILIP1L knockdown affected growth of lung cancer cells *in vivo*. As shown in Fig. 2G–J, knockdown of FILIP1L significantly enhanced growth of subcutaneous tumor xenografts in athymic nude mice. Interestingly, while H1944 cells grew faster than H441 cells *in vitro* (Fig. 2E and F; average time for cytokinesis completion was 132 and 213 minutes in control clones of H1944 and H441 cells, respectively), tumors from H1944 cells grew slower than those from H441 cells (Fig. 2G and I). As determined by a clinical pathologist (G. Riedlinger), tumors from FILIP1L-knockdown clones demonstrated higher grade (moderately to poorly differentiated; 7/8 tumors) than those from control clones (well to moderately differentiated; 6/8 tumors) of H1944 cells. Tumors from FILIP1L-knockdown clones demonstrated a significantly higher Ki67 index (Fig. 2K–M; higher magnification images are shown in insets of Fig. 2K and Supplementary Fig. S2B), suggesting more proliferation than those from control clones.

### FILIP1L Loss in Mouse Lung Induces Neoplastic Changes

**FIGURE 3:**
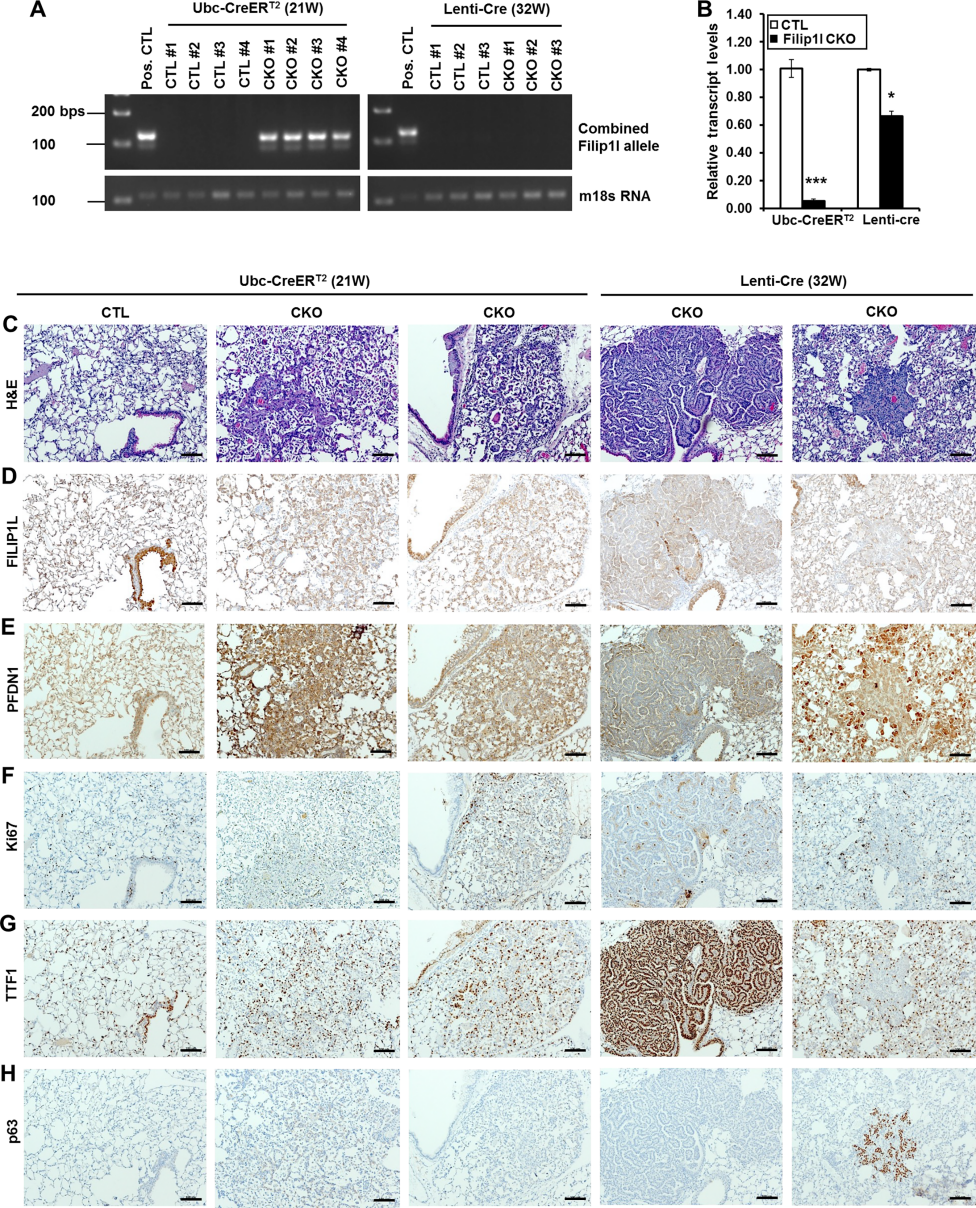
FILIP1L loss in mouse lung induces neoplastic changes. **A** and **B,** Littermate *Filip1l^fl/fl^* (CTL) and *Filip1l^fl/fl^; Ubc-CreER^T2^* (CKO) mice were treated with daily doses of tamoxifen (160 mg/kg) for 5 days and sacrificed at 21 weeks. C57BL6/J (CTL) and *Filip1l^fl/fl^* (CKO) mice were injected intratracheally with lentiviruses encoding Cre recombinase (Lenti-Cre) and sacrificed at 32 weeks. Genomic DNAs and total RNAs were purified from fixed lung tissues, followed by genotyping for combined *Filip1l*-floxed allele **(A)** and qRT-PCR for *Filip1l* mRNA levels **(B).** Genotyping for a housekeeping gene, *m18s RNA* is also shown for loading control. The *y* axis in **B** represents fold change over CTL mice, where each value was standardized with the housekeeping gene *β-actin* (3–4 mice each). * and *** indicate *P* < 0.05 and *P* < 0.001, respectively. **C–H,** Lungs were fixed, and stained with H&E **(C)**. They were also immunohistochemically stained for FILIP1L **(D)**, PFDN1 **(E)**, Ki67 **(F)**, TTF1 **(G)**, and p63 **(H)**. CTL derived from *Filip1l^fl/fl^* mice treated with tamoxifen. Scale bar = 100 μm.

To address the phenotypic consequence of *FILIP1L* gene inactivation in the lung, *Filip1l*-floxed mice, which we generated previously (18) were crossed with *Ubc-CreER^T2^* transgenic mice that express a TAM-regulated Cre protein (CreER^T2^) for deletion of *loxP*-containing alleles in whole body. Because we did not know which cell types in mouse lung will be affected following *Filip1l* knockout, we chose to use a whole body–inducible knockout model. We also attempted to knockout *Filip1l* in mouse lung in a tissue-specific manner by intratracheally administering lentiviruses encoding Cre recombinase (named as Lenti-Cre). To examine *Filip1l* gene deletion efficiency, we prepared both genomic DNA and total RNA from the fixed mouse lung tissues. We detected a robust band for the combined *Filip1l* allele in the *Filip1l* CKO mice (CKO) from *Ubc-CreER^T2^* group, but not from the Lenti-Cre group (Fig. 3A). From qPCR analysis, we observed *Filip1l* mRNA expression was reduced by approximately 20-fold and 1.5-fold in the CKO mice from *Ubc-CreER^T2^* group and Lenti-Cre group, respectively compared with their corresponding control mice (CTL; Fig. 3B).

Twenty-one weeks after TAM induction in CKO mice from the *Ubc-CreER^T2^* group, hematoxylin and eosin (H&E) staining demonstrated regions of atypical adenomatous hyperplasia, as evidenced by aberrant cell arrangements and irregular nuclei (Fig. 3C, second and third columns; higher magnification images are shown in Supplementary Fig. S3, second and third rows). Thirty-two weeks following lentivirus injection in CKO mice from the Lenti-Cre group, H&E staining demonstrated regions of adenomas (Fig. 3C, fourth and fifth columns; higher magnification images are shown in Supplementary Fig. S3, fourth and fifth rows). No adenomas were evident in CTL mice. *Filip1l* CKO mice were monitored up to a year; however, we have not observed adenocarcinoma formation. FILIP1L expression was reduced in CKO mice (Fig. 3D; higher magnification images from three additional adenomas are shown in Supplementary Fig. S4A and S4B). PFDN1 appeared to be increased in the areas of neoplastic changes in the lung where FILIP1L expression was reduced from CKO mice (Fig. 3E). These areas of neoplastic change demonstrated higher Ki67 index than in the normal lungs from CTL mice (Fig. 3F; Supplementary Fig. S3). Importantly, they demonstrated strong TTF1 (also known as NKX2-1) expression (Fig. 3G; Supplementary Fig. S3). The majority of the areas of neoplastic change were negative for p63, a LUSC marker; however, we occasionally found areas that stained positive for p63 (Fig. 3H, fifth columns). These lesions can be defined as adenosquamous differentiation, because they also contain approximately 15% TTF1-positive glandular cells. Collectively, these findings suggest that FILIP1L downregulation leads to neoplastic changes associated with adenocarcinoma differentiation, confirming the tumor suppressor function of FILIP1L in the lungs.

### FILIP1L Loss in Mouse Lung Induces Mucin Secretion, Collagen Fiber Deposits, and Immune Infiltration

**FIGURE 4:**
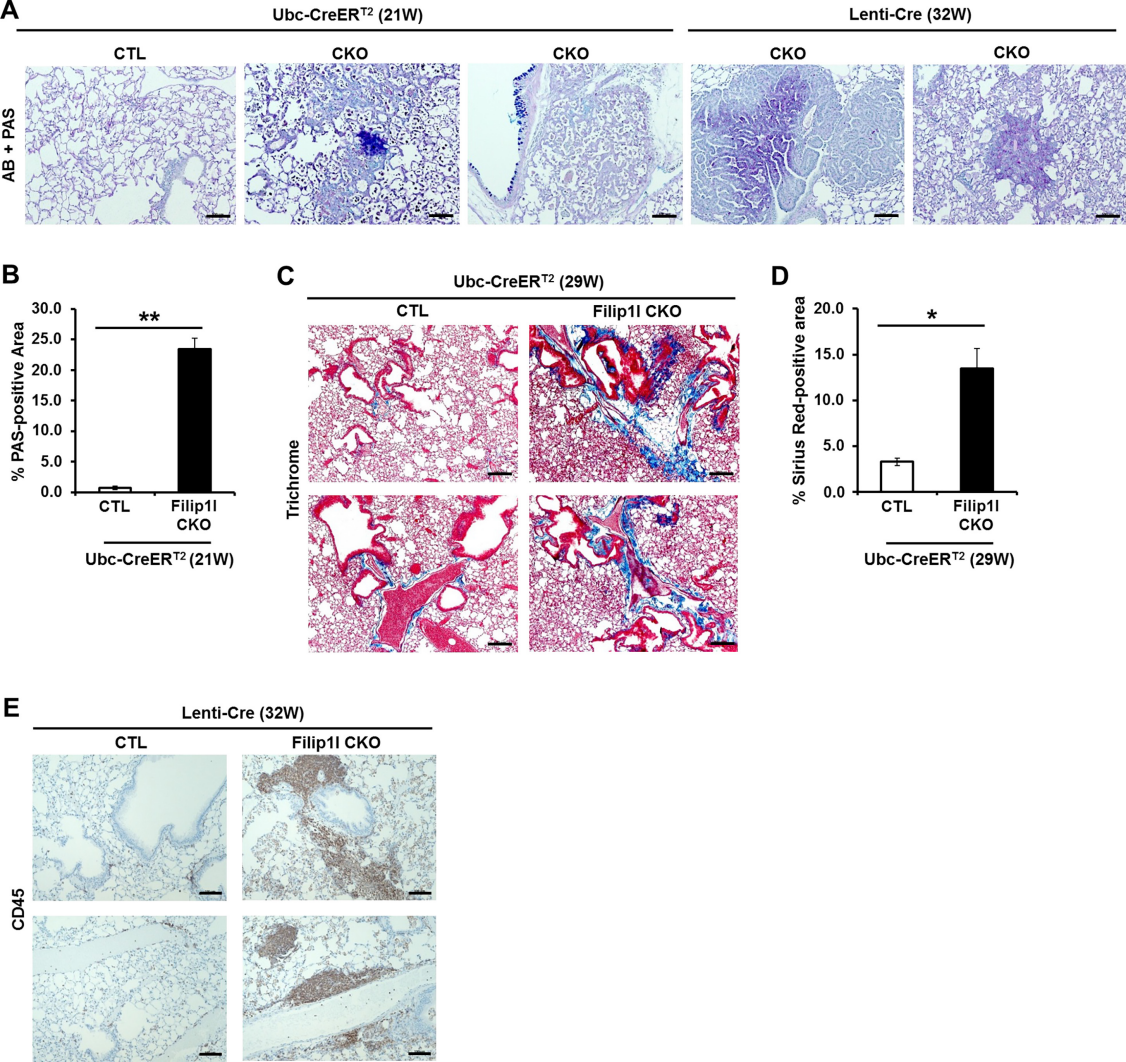
FILIP1L loss in mouse lung induces mucin secretion, collagen fiber deposits, and immune infiltration. **A** and **B,** Mucin secretion. **A,** The same regions imaged in Fig. 3C–H were stained with Alcian Blue and PAS. Blue and magenta color represents staining for acidic and neutral mucin proteins, respectively. Scale bar = 100 μm. **B,** Tamoxifen-treated *Filip1l^fl/fl^* (CTL) and *Filip1l^fl/fl^; Ubc-CreER^T2^* (CKO) mice were sacrificed at 21 weeks, and the lung tissues were subjected to PAS staining. Stitched images of PAS-stained lung sections are shown in Supplementary Fig. S5. PAS-stained area was quantified from the stitched images. Analysis excluded tracheal area and detailed quantification procedures were written in Supplementary Data. **C,** Tamoxifen-treated *Filip1l^fl/fl^* (CTL) and *Filip1l^fl/fl^; Ubc-CreER^T2^* (CKO) mice were sacrificed at 29 weeks, and the lung tissues were subjected to Trichrome staining. Blue color represents staining for collagen fibers. Scale bar = 100 μm. **D,** The same lung tissues used in **C** were subjected to Picro-Sirius Red staining. Stitched images of Picro-Sirius Red-stained lung sections are shown in Supplementary Fig. S6. Picro-Sirius Red-stained area was quantified from the stitched images. Detailed quantification procedures were written in Supplementary Data. **E,** Lenti-Cre–treated C57BL6/J (CTL) and *Filip1l^fl/fl^* (CKO) mice were sacrificed at 32 weeks, and the lung tissues were immunohistochemically stained for CD45. Scale bar = 100 μm. Stitched images of all the lung lobes from entire group (3 CTL and 3 *Filip1l* CKO mice) are shown in Supplementary Fig. S7. * and ** indicate *P* < 0.05 and *P* < 0.01, respectively.

We have recently shown that mucin hypersecretion was one of phenotypes in colon-specific *Filip1l* CKO mice (18). Mucin proteins are overexpressed in LUAD and their overexpression is significantly associated with enhanced metastasis and poor prognosis in NSCLC (27–29, 39, 40). Combined staining with Alcian blue and PAS on the lesions of neoplastic change demonstrated a considerable increase in mucin secretion compared with normal lungs (Fig. 4A). We consistently observed high mucin secretion throughout the lungs in the CKO mice from both *Ubc-CreER^T2^* and Lenti-Cre groups. Lungs from *Ubc-CreER^T2^* groups were stained with PAS and imaged with stitching, and PAS-positive areas were quantified (Supplementary Fig. S5). Mucin secretion was significantly increased in the lungs of the CKO mice compared with CTL mice (Fig. 4B).

Mucins are highly implicated in the process of pulmonary fibrosis [increased extracellular matrix (ECM) components in tumor microenvironment] (41). Increased ECM promotes cancer cell invasion, progression, and metastasis, and correlates with decreased survival in NSCLC (42, 43). FILIP1L knockdown was previously shown to increase ECM synthesis (44). Thus, we examined the expression of collagen fibers in the lungs of *Filip1l* CKO mice. Trichrome stain of the lungs from the *Ubc-CreER^T2^* group demonstrated a considerable increase in collagen fibers in the lungs of the CKO mice compared with CTL mice (Fig. 4C). To quantify the collagen fibers, we stained the lungs with Picro-Sirius Red, another specific stain for collagen fibers (Supplementary Fig. S6). Collagen fibers were significantly increased in the lungs of the CKO mice compared with CTL mice (Fig. 4D).

We also routinely observed high immune cell infiltration in the CKO mice from both *Ubc-CreER^T2^* and Lenti-Cre groups. Lungs from Lenti-Cre groups were stained for CD45, a marker for all immune cells except erythrocytes and platelets. As shown in Fig. 4E and Supplementary Fig. S7, a considerable number of immune cells was often located near the bronchioles/blood vessels in the lungs of CKO mice.

### FILIP1L Knockdown Enhances Syngeneic Allograft Tumor Growth *In Vivo*

**FIGURE 5:**
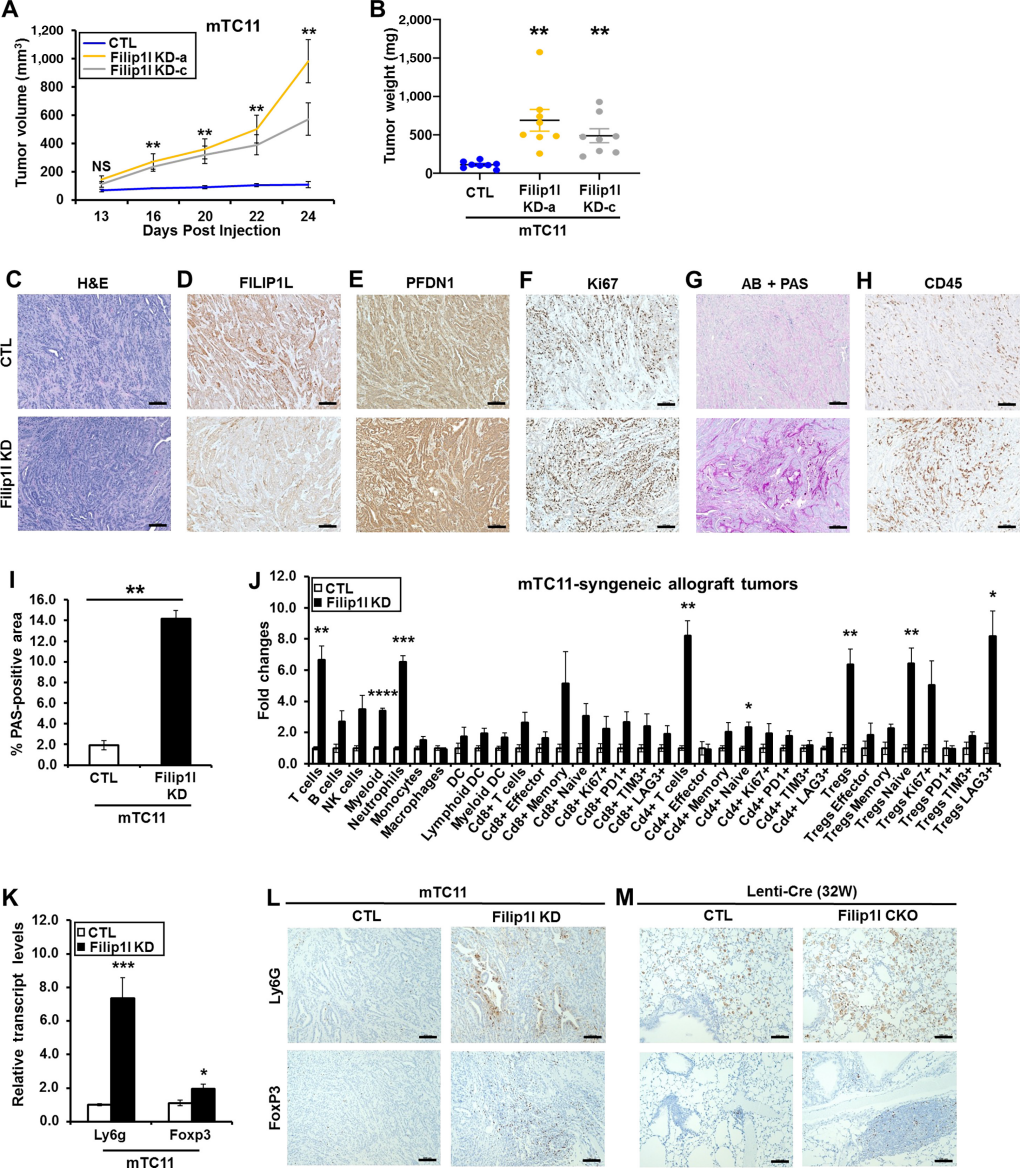
FILIP1L knockdown enhances syngeneic allograft tumor growth *in vivo*. mTC11 clones (1 × 10^6^) of either control or *Filip1l*-knockdown derivatives were subcutaneously injected into the C57BL6/J mice (8 mice per cell line). Tumor growth **(A)** was measured, every 2–3 days for a total of 24 days, as described in Fig. 2G, and tumor weights at the time of sacrifice **(B)** were measured. mTC11-syngeneic allograft tumors from either control or *Filip1l*-knockdown derivatives were fixed, and stained with H&E **(C)** as well as Alcian Blue and PAS **(G)**. They were also immunohistochemically stained for FILIP1L **(D)**, PFDN1 **(E)**, Ki67 **(F)**, and CD45 **(H)**. Scale bar = 100 μm. **I,** PAS-stained area in these tumors was quantified from the stitched images (3 CTL and 3 KD), as described in Fig. 4B. Stitched images of PAS-stained tumor sections are shown in Supplementary Fig. S8C and S8D. **J,** Fresh tumors from both control and *Filip1l*-knockdown groups were harvested at day 24 after subcutaneous injection. Dissociated cells were immunofluorescently stained with various markers for immune cells and subjected to FACS analysis. The *y* axis represents fold changes in *Filip1l*-knockdown tumors over CTL tumors for each cell type (five tumors each). **K,** Tumors from both control and *Filip1l*-knockdown groups were harvested at day 24 after subcutaneous injection, and snap frozen. mRNA levels of markers for neutrophils (*Ly6g*) and Tregs (*Foxp3*) in these frozen tumors were measured by qRT-PCR. The *y* axis represents fold changes in *Filip1l*-knockdown tumors over CTL tumors, where each value was standardized with the housekeeping gene *Rpl7* (6 tumors each). mTC11-syngeneic allograft tumors from either control or *Filip1l*-knockdown derivatives **(L)** and lungs from Lenti-Cre–treated either C57BL6/J (CTL) or *Filip1l^fl/fl^* (CKO) mice (as described in Fig. 4E; **M**) were immunohistochemically stained for Ly6G and FoxP3. Scale bar = 100 μm. *, **, ***, and **** indicate *P* < 0.05, *P* < 0.01, *P* < 0.001, and *P* < 0.0001, respectively.

To identify the effects of FILIP1L knockdown on lung tumor growth in an immune competent system, we utilized a mouse lung cancer cell line, mTC11 that harbors a *Kras^G12D^* mutation (30). Using two different constructs of lentiviral transduction, we knocked down FILIP1L in mTC11 cells. Immunoblotting confirmed that knockdown of FILIP1L resulted in increased PFDN1 expression (Supplementary Fig. S8A). Knockdown of FILIP1L significantly enhanced tumor growth compared with controls in both *Filip1l*-knockdown cell lines (Fig. 5A and B; Supplementary Fig. S8B). IHC staining confirmed decreased FILIP1L expression (Fig. 5D) and increased PFDN1 expression (Fig. 5E) in the tumors from *Filip1l*-knockdown clones. As determined by a clinical pathologist (G. Riedlinger), tumors from both CTL and *Filip1l*-knockdown clones demonstrated acinar pattern in the center and solid pattern in the edges. However, there were differences of tumor grade in the acinar area. While CTL tumors show well to moderate differentiation, *Filip1l*-knockdown tumors show more moderate differentiation, which was reflected in higher Ki67 index (Fig. 5F). In addition, *Filip1l*-knockdown tumors show considerable central necrosis that is an evidence of higher proliferation rate outpacing tumor blood supply [as shown in the stitched images of the tumors (Supplementary Fig. S8C and S8D)]. We also observed that the acinar area of *Filip1l*-knockdown tumors secrete considerably more mucins than CTL tumors as shown by PAS stain (Fig. 5G). From the PAS-stained stitched images (Supplementary Fig. S8C and S8D), mucin secretion was significantly increased in *Filip1l*-knockdown tumors compared with CTL tumors (Fig. 5I).

Having shown substantially more immune cell infiltration in the lungs from the CKO mice compared with CTL mice (Fig. 4E; Supplementary Fig. S7), we also observed more immune cells in *Filip1l*-knockdown tumors compared with CTL tumors in this syngeneic allograft model (Fig. 5H). We then asked which population of immune cells are increased following FILIP1L knockdown. Total immune cell pools from fresh tumors were stained with antibodies for various immune cell markers that were conjugated with fluorescent secondary antibodies (Supplementary Fig. S9A). Multiplexed cell pools were then subjected to flow cytometric analysis. The analysis scheme is outlined in Supplementary Fig. S9B. Representative FACS data and the quantified results are shown in Supplementary Fig. S9C and Fig. 5J, respectively. The following immune cell types were significantly increased in *Filip1l*-knockdown tumors compared with CTL tumors: (i) Myeloid cells; (ii) Neutrophils among myeloid population; (iii) T cells among lymphocyte population; (iv) CD4^+^ T cells among T-cell population; (v) CD4^+^ naïve cells and Tregs among CD4^+^ population; (vi) Naïve and LAG3+ cells among Tregs population. Neutrophils and Tregs were previously shown to be potential immune suppressive factors in NSCLC (45). In addition, neutrophil transcript signature was the strongest predictor of mortality of any immune cell types in NSCLC (46). From qPCR analysis, mRNA expression for *Ly6 g* [neutrophil marker (47)] and *Foxp3* [Treg marker (48)] was increased by approximately 7.3-fold and 2-fold, respectively in *Filip1l*-knockdown tumors compared to CTL tumors (Fig. 5K). IHC staining confirmed increased expression of Ly6G and FoxP3 in *Filip1l*-knockdown tumors compared with CTL tumors (Fig. 5L). Importantly, both Ly6G and FoxP3 were also considerably increased in the lungs of the *Filip1l* CKO mice compared with CTL mice (Fig. 5M).

### FILIP1L Knockdown Promotes Signaling Pathways Associated with Wnt/β-Catenin Signaling

**FIGURE 6:**
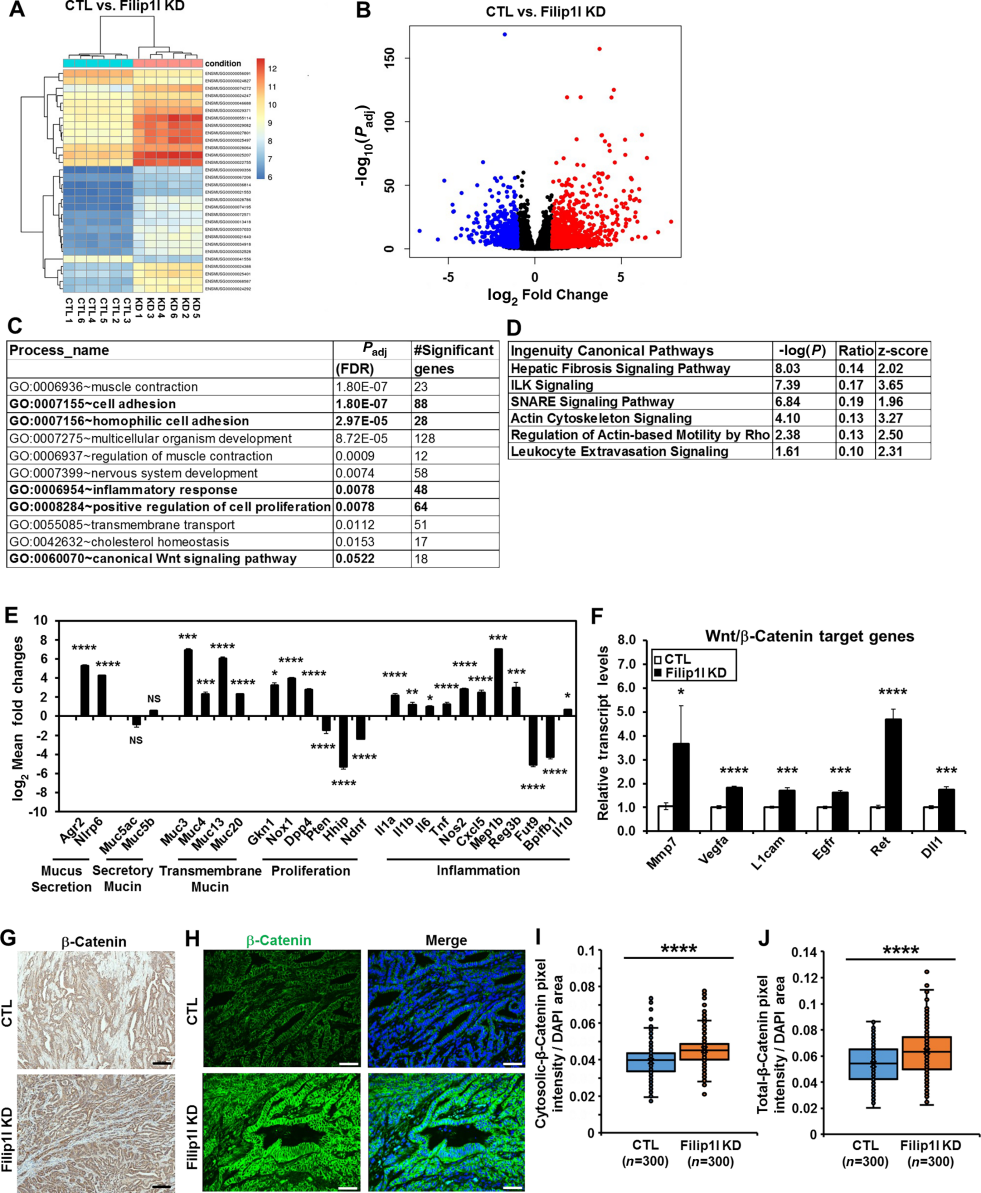
FILIP1L knockdown promotes signaling pathways associated with Wnt/β-catenin signaling. **A–D,** RNA-seq analysis was performed on the frozen mTC11-syngeneic allograft tumors between control and *Filip1l*-knockdown groups (as described in Fig. 5K; six tumors each). **A,** Biclustering heatmap clusters both the samples and the genes for the top 30 differentially expressed genes. Ensembl gene ID of the 30 genes is shown. Red and blue color indicate higher and lower relative expression, respectively. **B,** Volcano plot maps fold changes against *P* values, which highlight the set of significantly differentially expressed genes. *x* axis represents log_2_ fold changes. *y* axis represents the Benjamini–Hochberg adjusted *P* values shown in negative log_10_ values. Upregulated and downregulated genes are shown in red and blue dots, respectively. log_2_ fold changes between −1 and 1 were considered nonsignificant, and these genes are shown in black dots. **C,** GO groups associated with *Filip1l*-knockdown tumors were identified by GO analysis using GeneSCF software. Significant GO groups by FDR-adjusted *P* values are shown. **D,** Canonical pathways upregulated in *Filip1l*-knockdown tumors were identified by Ingenuity Pathway Analysis (IPA) software. Significant canonical pathways by *P* values generated in IPA software are shown. Positive z-score indicates upregulated pathway. Both GO and canonical pathway analysis were performed on the statistically significant set of genes. mRNA levels of markers for mucus secretion, mucin, proliferation, and inflammation **(E)** as well as Wnt/β-catenin target genes **(F)** were measured by qRT-PCR on mTC11 tumors from both control and *Filip1l*-knockdown groups (as prepared in Fig. 5K). The *y* axis represents log_2_ mean fold changes **(E)** or fold changes **(F)** in *Filip1l*-knockdown tumors over CTL tumors, where each value was standardized with the housekeeping gene *Rpl7* (six tumors each). **G–J,** β-catenin expression in mTC11 syngeneic allograft tumors. Tumor sections described in Fig. 5C–H were stained for β-catenin both immunohistochemically (**G**; Scale bar = 100 μm) and immunofluorescently (**H**; green color; Scale bar = 50 μm). Nuclei were counterstained with DAPI (blue), and merged fluorescent images are also shown **(H)**. Cytosolic **(I)** or total **(J)** β-catenin-positive areas from the immunofluorescence staining were quantified. One hundred random fields per mouse were quantified (3 mice each). *, **, ***, and **** indicate *P* < 0.05, *P* < 0.01, *P* < 0.001 and *P* < 0.0001, respectively.

To identify the downstream pathways affected by FILIP1L knockdown, we performed RNA-seq from syngeneic allograft tumors. Tumors from CTL and *Filip1l*-knockdown groups (six tumors each) demonstrated a clear segregation between groups (Fig. 6A; Supplementary Fig. S10A and S10B). A volcano plot demonstrated a significant differential gene expression between CTL and *Filip1l*-knockdown groups (Fig. 6B). Using GeneSCF software, we then performed GO analysis on these differentially expressed genes. As shown in Fig. 6C, signaling pathways such as muscle contraction, cell adhesion, inflammation, and cell proliferation were significantly increased in *Filip1l*-knockdown tumors (entire GO list is shown in Supplementary Table S1). We further performed Ingenuity Pathway Analysis. Canonical pathways including hepatic fibrosis and leukocyte extravasation were significantly increased as indicated by positive z-scores (Fig. 6D; entire pathway list is shown in Supplementary Table S2). From Ingenuity Pathway Analysis, we also analyzed “diseases and functions” categories. Muscle contraction and various cancer formation pathways were shown as ranked by *P* value (Supplementary Table S3). Among them, the predicted disease and/or function with the most significance and the highest z-score was “cancer of secretory structure.” We then validated the RNA-seq results using qPCR analysis. In line with the findings described earlier (Figs. 4A and B, 5G, and 5I), molecules involved in mucus secretion, *Agr2* and *Nlrp6* (49, 50) were significantly upregulated in *Filip1l*-knockdown tumors (Fig. 6E). However, the expression of two major secretory airway mucins, *Muc5ac* and *Muc5b* (24) was not changed transcriptionally. Importantly, several transmembrane mucins such as *Muc3*, *Muc4*, *Muc13,* and *Muc20* (24) were highly increased in *Filip1l*-knockdown tumors (Fig. 6E). Molecules known to promote [*Gkn1*, *Nox1,* and *Dpp4* (51–53)] and inhibit [*Pten*, *Hhip,* and *Ndnf* (54–56)] cell proliferation were increased and decreased, respectively in *Filip1l*-knockdown tumors (Fig. 6E). Molecules known to promote [*Il1a*, *Il1b*, *Il6*, *Tnf*, *Nos2*, *Cxcl5*, *Mep1b,* and *Reg3b* (57–62)] and reduce [*Fut9* and *Bpifb1* (63, 64)] inflammation were increased and decreased, respectively in *Filip1l*-knockdown tumors (Fig. 6E). *Il10*, an anti-inflammatory cytokine that is highly upregulated in multiple immune cell lineages including activated Foxp3^+^ Tregs (65, 66), was increased. *Nlrp6* and *Dpp4* were also shown to increase inflammation (50, 53). *Nox1*, *Tnf*, *Mep1b,* and *Reg3b* were also implicated in ECM organization and fibrosis (52, 62, 67, 68).

From the ontology analysis of RNA-seq data, we demonstrate here that activation of the canonical Wnt/β-catenin signaling pathway approached a significant difference (Fig. 6C). Furthermore, upregulated pathways such as cell adhesion, proliferation, inflammation, and fibrosis in *Filip1l*-knockdown syngeneic allograft tumors (Fig. 6C and D) have been shown to be closely associated with the activation of Wnt/β-catenin signaling (69). Thus, we examined whether the target genes for Wnt/β-catenin signaling pathway are activated in *Filip1l*-knockdown tumors. As shown in Fig. 6F, various Wnt/β-catenin target genes such as *Mmp7*, *Vegfa*, *L1cam*, *Egfr*, *Ret,* and *Dll1* (70–75) were significantly increased in *Filip1l*-knockdown tumors compared with CTL tumors. In addition, the Wnt/β-catenin target genes shown in Fig. 6E such as *Muc4*, *Nos2*, *Il6,* and *Il10* (69, 76–78) were also activated. We then stained these tumors for active (triple non-phospho) β-catenin protein. Considerably more active β-catenin was detected in *Filip1l*-knockdown tumors compared with CTL tumors using both IHC (Fig. 6G) and immunoflurorescence (Fig. 6H) staining. While the majority of staining was detected as membranous, most of the cells in *Filip1l*-knockdown tumors also demonstrated a diffusive cytosolic staining indicative of activated β-catenin (79). Indeed, the amount of activated β-catenin in the cytosol (Fig. 6I) as well as in total cells (Fig. 6J) was significantly increased in *Filip1l*-knockdown tumors compared with CTL tumors. Nuclear localization of β-catenin, a hallmark of activation of Wnt signaling, was rarely observed. It was previously shown that nuclear β-catenin was observed only in a subpopulation of cells from the tumors that had progressed from adenoma to adenocarcinoma in genetically engineered mouse models of lung cancer (80). Thus, these findings collectively suggest that FILIP1L downregulation in lung cancer is associated with the phenotypes related to the Wnt/β-catenin signaling pathway.

## Discussion

*FILIP1L* expression is downregulated in the majority of human malignancies including NSCLC. From the 23 lung cancer databases in the cBioPortal search, the somatic mutation frequency for *FILIP1L* gene is only 0.9% and the majority are missense mutations. We previously showed that FILIP1L is downregulated by promoter methylation in cancer cell lines of various histologies including lung, ovarian, colon, breast, and pancreas (14, 16). Promoter methylation-associated FILIP1L downregulation was also implicated in human cancer tissues such as ovarian, prostate, and cutaneous squamous cell carcinoma (14, 81, 82). We show here that *FILIP1L* is downregulated by promoter methylation in both LUAD and LUSC, and that repression of *FILIP1L* in either of these tumors correlates with decreased patient survival.

Cigarette smoking is the major cause of lung cancer deaths (1, 2), and mutational signatures associated with cigarette smoking are well established in human cancer (83). Cigarette smoking has been linked to aberrant DNA methylation in lung cancers (8, 9, 12). We previously showed that *FILIP1L* is downregulated by promoter methylation in normal human respiratory epithelial cells following short-term exposure to tobacco condensates (13). In our current experiments, *FILIP1L* downregulation was highly associated with *FILIP1L* promoter methylation in cultured LUAD cells, and *FILIP1L* repression as well as *FILIP1L* promoter methylation in LUAD specimens were significantly associated with cigarette smoking. Whereas *FILIP1L* downregulation also correlated with *FILIP1L* promoter methylation in LUSC specimens, neither appeared to be associated with cigarette smoking, suggesting that factors other than cigarettes contribute to epigenetic repression of this tumor suppressor gene in these neoplasms.

Our current experiments demonstrated that targeted knockout of FILIP1L leads to pulmonary adenoma formation in mice; these observations suggest that repression of *FILIP1L* is an important event during initiation or early progression of LUADs. In contrast, our bioinformatics analysis of bulk RNA-seq data demonstrated that repression of *FILIP1L* was more evident in locally advanced or metastatic LUAD, suggesting a greater impact of this event on later stages of progression in LUAD. In addition, comprehensive studies involving cell lines, murine models, and human specimens are necessary to more fully define the timing and mechanisms of FILIP1L downregulation during LUAD development, and to determine the potential utility of *FILIP1L* promoter methylation as a biomarker of aggressive phenotype and poor prognosis in patients with these neoplasms. Although knocking out FILIP1L in mouse lung led to pulmonary adenoma formation, it was not sufficient to induce adenocarcinomas. Further studies are warranted to test whether these adenomas progress to adenocarcinomas if the *Filip1l* CKO mice were treated with cigarette smoke or other environmental carcinogens.

Epithelial integrity is maintained by the cytoskeleton and through cell adhesion. FILIP1L regulates proteasome-dependent degradation of PFDN1, and increased PFDN1, caused by downregulation of FILIP1L, drives mucin secretion in colon cancer (18). PFDN1 is a molecular chaperone of a six subunit–prefoldin complex that facilitates proper folding of key cytoskeletal components such as actin and tubulins (84). Altered expression of prefoldin proteins leads to protein misfolding and aggregation, resulting in impaired protein homeostasis (proteostasis) that can drive various pathologic conditions including cancer (84). PFDN1 is overexpressed in multiple cancer types including lung, colon, and gastric cancer, and its overexpression is associated with poor prognosis in colon cancer and NSCLC (18, 21–23, 85, 86). Overexpressed PFDN1 promotes EMT, xenograft tumor formation, and metastasis in lung cancer cells (23, 85). Interestingly, upregulated PFDN1 was shown to activate Wnt/β-catenin signaling-mediated EMT that facilitates cell migration, invasion, and metastasis in gastric cancer (21). We previously demonstrated that FILIP1L knockdown increased the active β-catenin pool, thereby activating canonical Wnt/β-catenin signaling pathways in ovarian cancer (15, 19). We show here that FILIP1L knockdown and the resultant increase in PFDN1 led to upregulate the Wnt/β-catenin signaling pathway in *Filip1l*-knockdown syngeneic allograft tumors.

Pathways regulating mucin expression are overexpressed especially in LUAD (87, 88). Mucin proteins such as MUC5AC, MUC5B, MUC1, MUC3, MUC4, and MUC13 are significantly overexpressed in LUAD patient samples (27, 39, 40). Aberrant overexpression and glycosylation of various mucin proteins have been associated with immune modulation and metastatic progression in various adenocarcinomas including LUAD (89). We show here that, along with secreted mucins, transmembrane mucins such as *Muc3*, *Muc4*, *Muc13,* and *Muc20* were highly increased in *Filip1l*-knockdown syngeneic allograft tumors. MUC4 and MUC13 are shown to contribute to carcinogenesis under inflammatory conditions (90). Fibrosis is a common pathology of chronic inflammation in many organs, including lungs and liver (69). Both secreted and transmembrane mucins are highly implicated in the process of pulmonary fibrosis (41). A polymorphism in the promoter of MUC5B is strongly associated with risk of developing pulmonary fibrosis (24), and MUC5B overexpression enhanced pulmonary fibrosis in a mouse model (91). Increased and cross-linked ECM promotes cancer cell invasion, progression, and metastasis in NSCLC (42). High stroma-tumor ratio (≥50% stroma) correlates with decreased survival in NSCLC (43). Presence of type I collagen results in decreased progression-free survival in patients with LUAD (92). In this study, downregulation of FILIP1L leads to a significant increase in accumulation of collagen fibers in the lungs of the *Filip1l* CKO mice compared with control mice. In addition, both WNT/β-catenin and hepatic fibrosis pathways were significantly increased in *Filip1l*-knockdown syngeneic allograft tumors. Activated Wnt/β-catenin signaling was shown to upregulate pathways such as cell adhesion, proliferation, inflammation, and fibrosis (69). Thus, findings in this study suggest that the observed phenotypes following FILIP1L knockdown such as mucin secretion, inflammation, and fibrosis are attributed to activated Wnt/β-catenin signaling.

A gene signature of invasive mucinous adenocarcinoma of the lung, which includes transcription factors (FOXA3, SPDEF, and HNF4A) and mucin proteins (MUC5AC, MUC5B, and MUC3) has been identified (26). FOXA3 and SPDEF induce MUC5AC and MUC5B, while HNF4A induces MUC3 in human lung cancer cells harboring a *KRAS* mutation. *KRAS* mutations are the most frequent genetic alterations seen in invasive mucinous adenocarcinoma (40%–62%) followed by *NRG1* fusion (7%–27%; refs. 93, 94). Knockdown of anti-mucous transcription factor, NKX2-1 (also known as TTF1) in *Kras^G12D^* induces mucinous adenocarcinoma of the lung in a murine model (93). Knocking out FILIP1L in mouse lungs led to TTF1-positive adenoma formation. Although TTF1 is shown to be absent in invasive mucinous adenocarcinoma of the lung (26), these neoplastic lesions in the lungs of FILIP1L-knockout mouse demonstrated strong mucin secretion. In fact, mucin secretion was prevalent in the lung parenchyma. When we knocked down FILIP1L in mutant *Kras*-harboring mTC11 cells, the resultant syngeneic allograft tumors demonstrated a strong mucin secretion along with significantly increased transmembrane mucins such as *Muc3*. Thus, it will be worthwhile to evaluate whether FILIP1L knockout in a mutant *Kras* background will result in mucinous adenoma and/or adenocarcinoma formation in mouse lungs. These results will also need to be validated in additional syngeneic mouse models harboring different *Kras* mutations or other genomic subtypes, as well as in a large panel of human LUAD tumors.

In summary, we have shown that a tumor suppressor *FILIP1L* is downregulated in the majority of human cancer types. In LUAD, its downregulation through promoter methylation is attributable at least in part to cigarette smoking. FILIP1L knockdown and the resultant PFDN1 increase lead to increased mucin secretion and/or overexpression in mouse lung as well as lung cancer cells, possibly generating a niche for lung cancer progression through increased inflammation and fibrosis. FILIP1L knockdown also leads to upregulated Wnt/β-catenin signaling, which could be responsible for the observed phenotypes such as increased proliferation, inflammation, and fibrosis. Collectively, these results strongly suggest that downregulation of FILIP1L is clinically relevant in LUAD and warrant further efforts to evaluate pharmacologic regimens that either directly or indirectly restore FILIP1L-mediated gene regulation for the treatment of these neoplasms.

## Supplementary Material

Supplementary Table S1 Supplementary Table S1

Supplementary Table S2 Supplementary Table S2

Supplementary Table S3 Supplementary Table S3

Supplementary Figures S1-S10 and Supplementary Information Supplementary Figures and Supplementary information
