# Supplementary material for: Smoking-associated Downregulation of FILIP1L Enhances Lung Adenocarcinoma Progression Through Mucin Production, Inflammation, and Fibrosis
Source: Cancer Res Commun. 2022 Oct 18;2(10):1197–213. doi: 10.1158/2767-9764.CRC-22-0233 (PMC9973389; doi:10.1158/2767-9764.CRC-22-0233)
Supplement: Supplementary Figures S1-S10 and Supplementary Information — Supplementary Figures and Supplementary information [file crc-22-0233-s04.pdf]

Suppl. Fig. S1

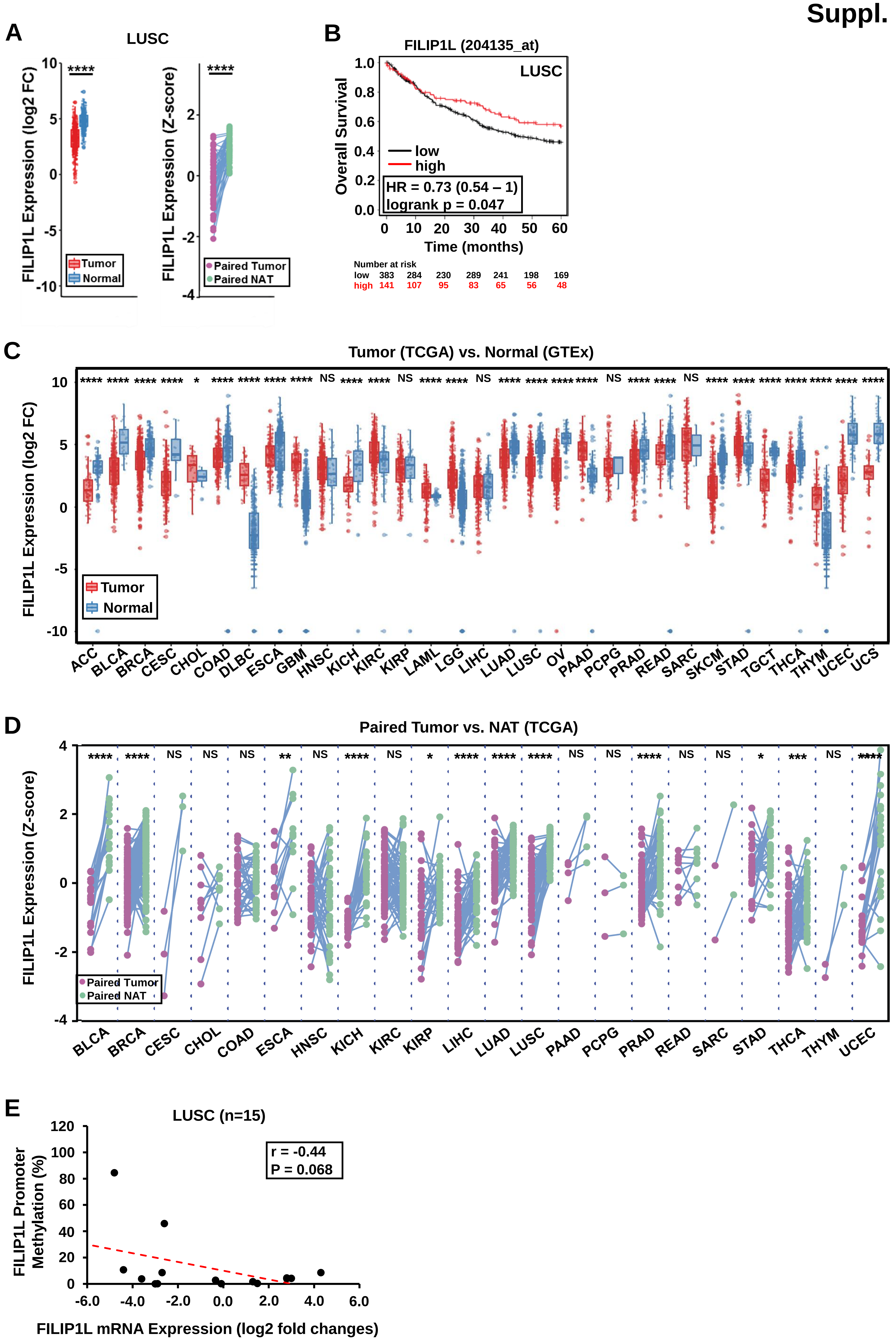
A
LUSC
FILIP1L Expression (log2 FC)
****
Tumor
Normal
FILIP1L Expression (Z-score)
****
Paired Tumor
Paired NAT
B
FILIP1L (204135_at)
LUSC
Overall Survival
low
high
HR = 0.73 (0.54 – 1)
logrank p = 0.047
Time (months)
Number at risk
low 383 284 230 289 241 198 169
high 141 107 95 83 65 56 48
C
Tumor (TCGA) vs. Normal (GTEx)
FILIP1L Expression (log2 FC)
Tumor
Normal
ACC BLCA BRCA CESC CHOL COAD DLBC ESCA GBM HNSC KICH KIRC KIRP LAML LGG LIHC LUAD LUSC OV PAAD PCPG PRAD READ SARC SKCM STAD TGCT THCA THYM UCEC UCS
D
Paired Tumor vs. NAT (TCGA)
FILIP1L Expression (Z-score)
Paired Tumor
Paired NAT
BLCA BRCA CESC CHOL COAD ESCA HNSC KICH KIRC KIRP LIHC LUAD LUSC PAAD PCPG PRAD READ SARC STAD THCA THYM UCEC
E
LUSC (n=15)
FILIP1L Promoter Methylation (%)
r = -0.44
P = 0.068
FILIP1L mRNA Expression (log2 fold changes)

Suppl. Fig. S1

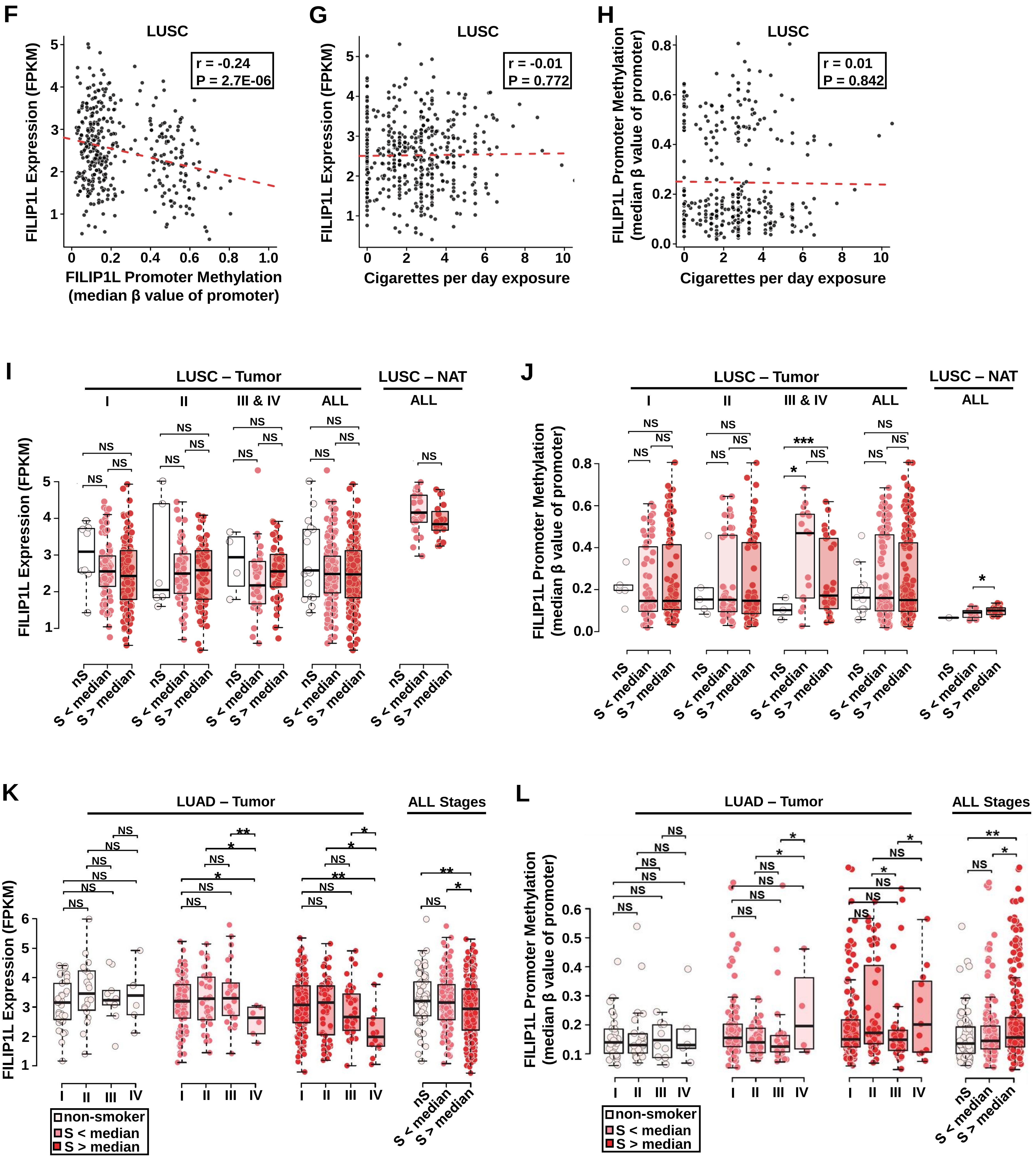
F
LUSC
r = -0.24
P = 2.7E-06
FILIP1L Expression (FPKM)
FILIP1L Promoter Methylation
(median β value of promoter)
G
LUSC
r = -0.01
P = 0.772
FILIP1L Expression (FPKM)
Cigarettes per day exposure
H
LUSC
r = 0.01
P = 0.842
FILIP1L Promoter Methylation
(median β value of promoter)
Cigarettes per day exposure
I
LUSC – Tumor
LUSC – NAT
I
II
III & IV
ALL
ALL
FILIP1L Expression (FPKM)
nS
S < median
S > median
J
LUSC – Tumor
LUSC – NAT
FILIP1L Promoter Methylation
(median β value of promoter)
K
LUAD – Tumor
ALL Stages
FILIP1L Expression (FPKM)
non-smoker
S < median
S > median
L
LUAD – Tumor
ALL Stages
FILIP1L Promoter Methylation
(median β value of promoter)
non-smoker
S < median
S > median

Supplementary Figure S1. (A-B) Experimental details were followed as described in **Figure 1A-B**. **(A)** FILIP1L mRNA expression between tumor and normal tissues from LUSC patients were compared. Plots shown are comparison between tumors and normal lungs (**Left panel**) or between paired tumors and NATs (**Right panel**). **(B)** Overall survival of LUSC patients whose FILIP1L expression was either low or high was analyzed in Kaplan–Meier plots. Cutoff values of 1007 were determined by auto select best cutoff option. Using the selected parameters, the analysis was run on 524 patients. **(C-D)** FILIP1L mRNA expression between tumor and normal tissues from various cancer types were compared. Plots shown are comparison between tumors and normal lungs **(C)** or between paired tumors and NATs **(D)**. The followings are TCGA Study Abbreviations (https://gdc.cancer.gov/resources-tcga-users/tcga-code-tables/tcga-study-abbreviations). **ACC**: Adrenocortical carcinoma; **BLCA**: Bladder Urothelial Carcinoma; **BRCA**: Breast invasive carcinoma; **CESC**: Cervical squamous cell carcinoma and endocervical adenocarcinoma; **CHOL**: Cholangiocarcinoma; **COAD**: Colon adenocarcinoma; **DLBC**: Lymphoid Neoplasm Diffuse Large B-cell Lymphoma; **ESCA**: Esophageal carcinoma; **GBM**: Glioblastoma multiforme; **HNSC**: Head and Neck squamous cell carcinoma; **KICH**: Kidney Chromophobe; **KIRC**: Kidney renal clear cell carcinoma; **KIRP**: Kidney renal papillary cell carcinoma; **LAML**: Acute Myeloid Leukemia; **LGG**: Brain Lower Grade Glioma; **LIHC**: Liver hepatocellular carcinoma; **LUAD**: Lung adenocarcinoma; **LUSC**: Lung squamous cell carcinoma; **OV**: Ovarian serous cystadenocarcinoma; **PAAD**: Pancreatic adenocarcinoma; **PCPG**: Pheochromocytoma and Paraganglioma; **PRAD**: Prostate adenocarcinoma; **READ**: Rectum adenocarcinoma; **SARC**: Sarcoma; **SKCM**: Skin Cutaneous Melanoma; **STAD**: Stomach adenocarcinoma; **TGCT**: Testicular Germ Cell Tumors; **THCA**: Thyroid carcinoma; **THYM**: Thymoma; **UCEC**: Uterine Corpus Endometrial Carcinoma; **UCS**: Uterine Carcinosarcoma. **(E)** Experimental details were followed as described in **Figure 1C**. Relationship between FILIP1L mRNA expression (*x* axis) and FILIP1L promoter methylation (*y* axis) is shown in cell lines derived from LUSC (P values shown are by Spearman's rank correlation method). The *x* axis represents FILIP1L mRNA expression of log2 fold changes from RNA-Seq data. The *y* axis represents percent methylation of the average overall methylation for all available CG sites in FILIP1L promoter. **(F-H)** LUSC samples from TCGA databases were analyzed for the relationship between **(F)** FILIP1L promoter methylation (*x* axis; median β value of FILIP1L promoter methylation) and FILIP1L mRNA expression (*y* axis; FPKM values from RNA-Seq data); **(G)** the daily amount of cigarette exposures (x axis) and FILIP1L mRNA expression (y axis); **(H)** the daily amount of cigarette exposures (x axis) and FILIP1L promoter methylation (y axis). **(I-J) (I)** FILIP1L mRNA expression or **(J)** FILIP1L promoter methylation was compared between non-smokers (nS), smokers exposed less than the median of tobacco dose (S < median) and smokers exposed more than the median of tobacco dose (S > median) in the stage I-IV tumors of LUSC patients. Comparison in the samples with all stages and NATs are also shown. Note that the plot for nS group in LUSC-NAT in panel **I** is missing due to non-availability of sample. **(K-L) (K)** FILIP1L mRNA expression or **(L)** FILIP1L promoter methylation was compared among different stage tumors from LUAD patients of non-smokers (nS), smokers exposed less than the median of tobacco dose (S < median) and smokers exposed more than the median of tobacco dose (S > median). *, **, *** and **** indicate P<0.05, P<0.01, P<0.001 and P<0.0001, respectively.

Suppl. Fig. S2

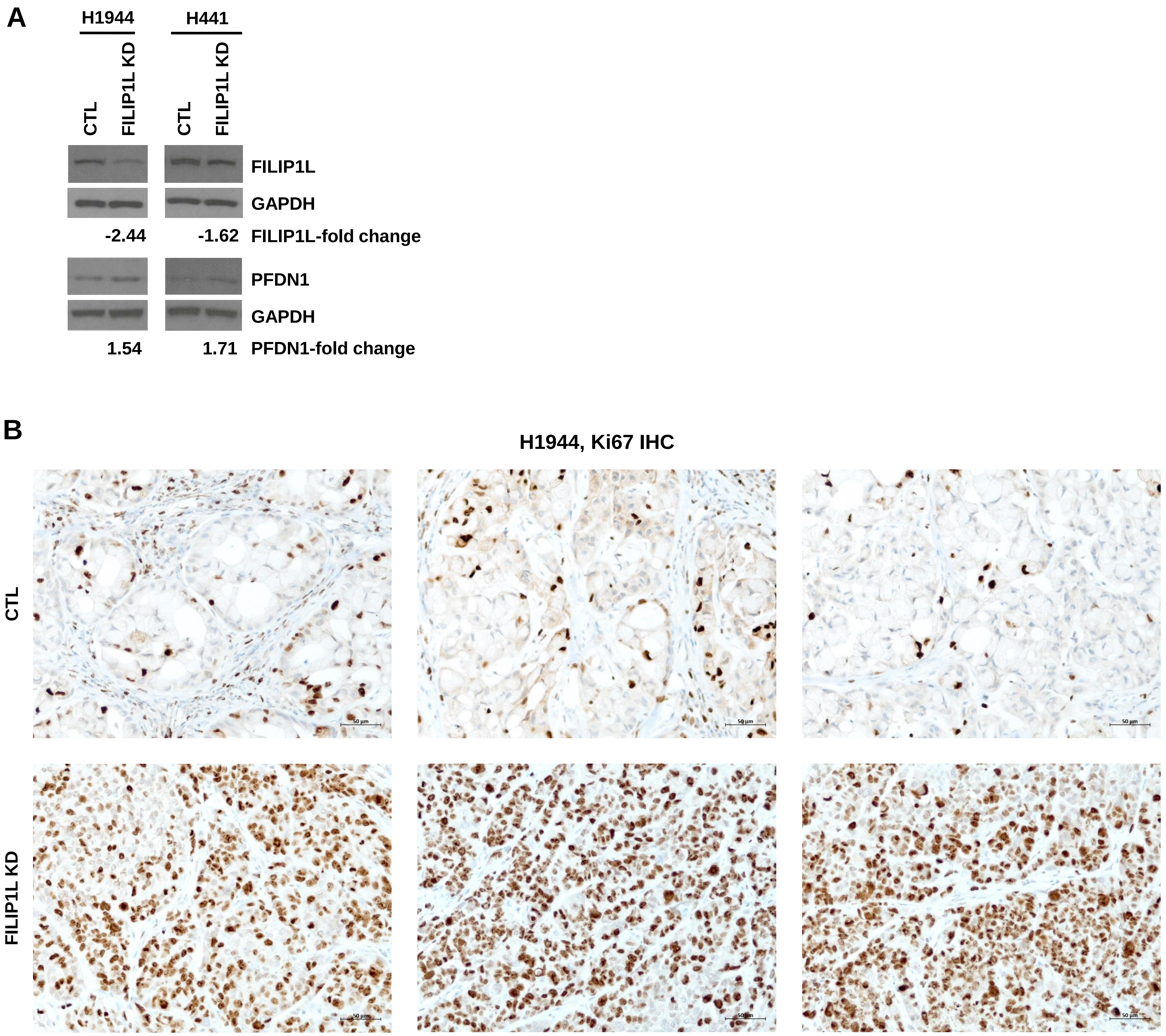

Supplementary Figure S2. FILIP1L knockdown was achieved by stable expression of Lentiviral shRNA in FILIP1L-high H1944 and H441 lung cancer cells. Pooled shRNAs from 4 different sequences for FILIP1L were used. Control clones were made with scrambled shRNA. **(A)** FILIP1L, PFDN1 and GAPDH control were detected by immunoblotting. By densitometric quantification, the ratio of FILIP1L/GAPDH and PFDN1/GAPDH was determined. Fold changes of either FILIP1L or PFDN1 in FILIP1L-knockdown (KD) clones over control (CTL) clones were calculated by dividing the ratio of either FILIP1L/GAPDH or PFDN1/GAPDH (KD/CTL). **(B)** Xenograft tumors from either control or FILIP1L-knockdown clones of H1944 were fixed and immunohistochemically stained for Ki67. Magnified images of Figure 2K are shown. Scale bar = 50 µm.

Suppl. Fig. S3

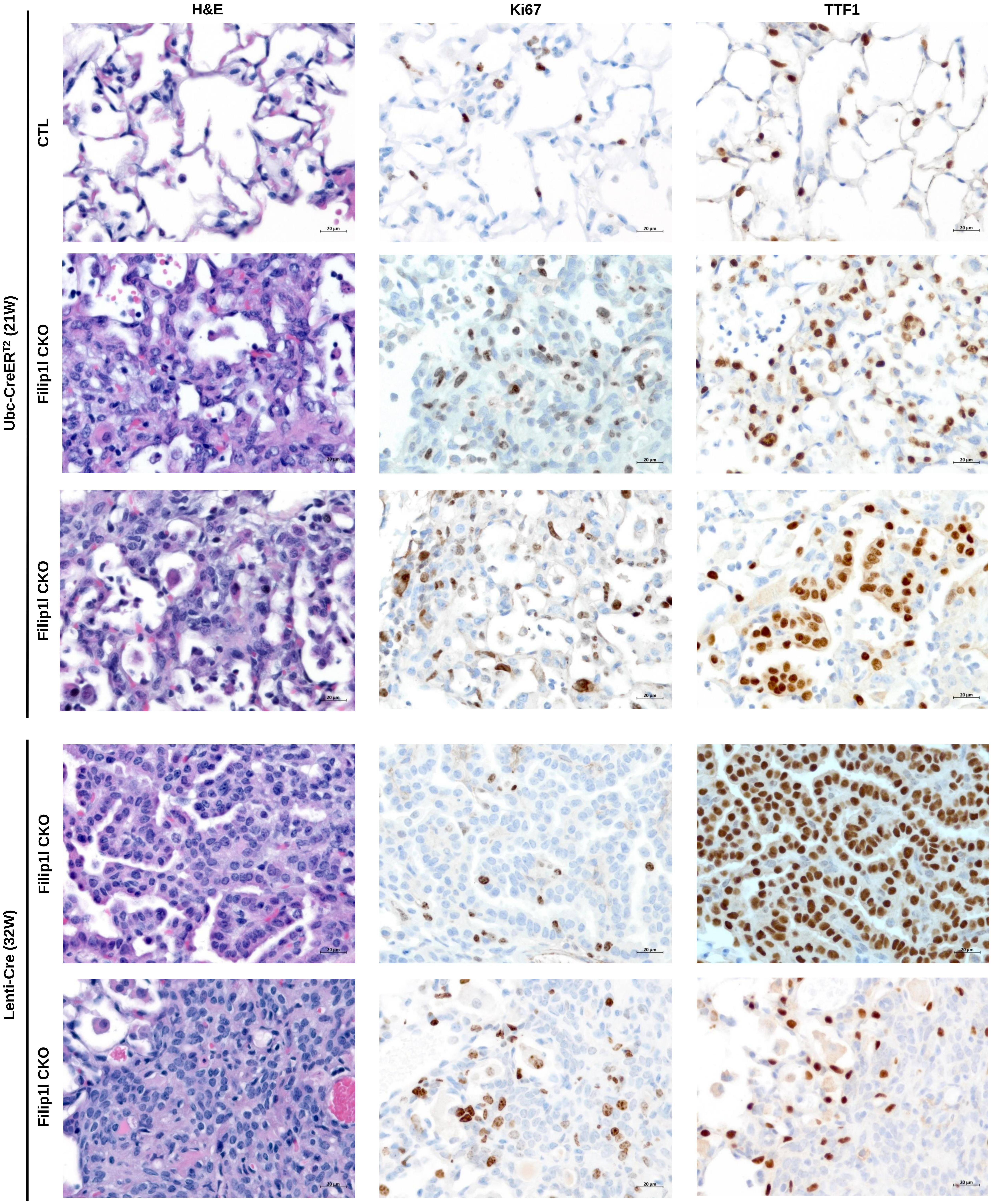

Supplementary Figure S3. Lung tissues from tamoxifen-treated Filip1l$^{fl/fl}$ (CTL) and Filip1l$^{fl/fl}$; Ubc-CreER^{T2} (CKO) mice sacrificed at 21 weeks, as well as Lenti-Cre-treated C57BL6/J (CTL) and Filip1l$^{fl/fl}$ (CKO) mice sacrificed at 32 weeks, were fixed and stained with H&E. They were also immunohistochemically stained for Ki67 and TTF1. Magnified images of Figure 3C, F and G are shown. Scale bar = 20 µm.

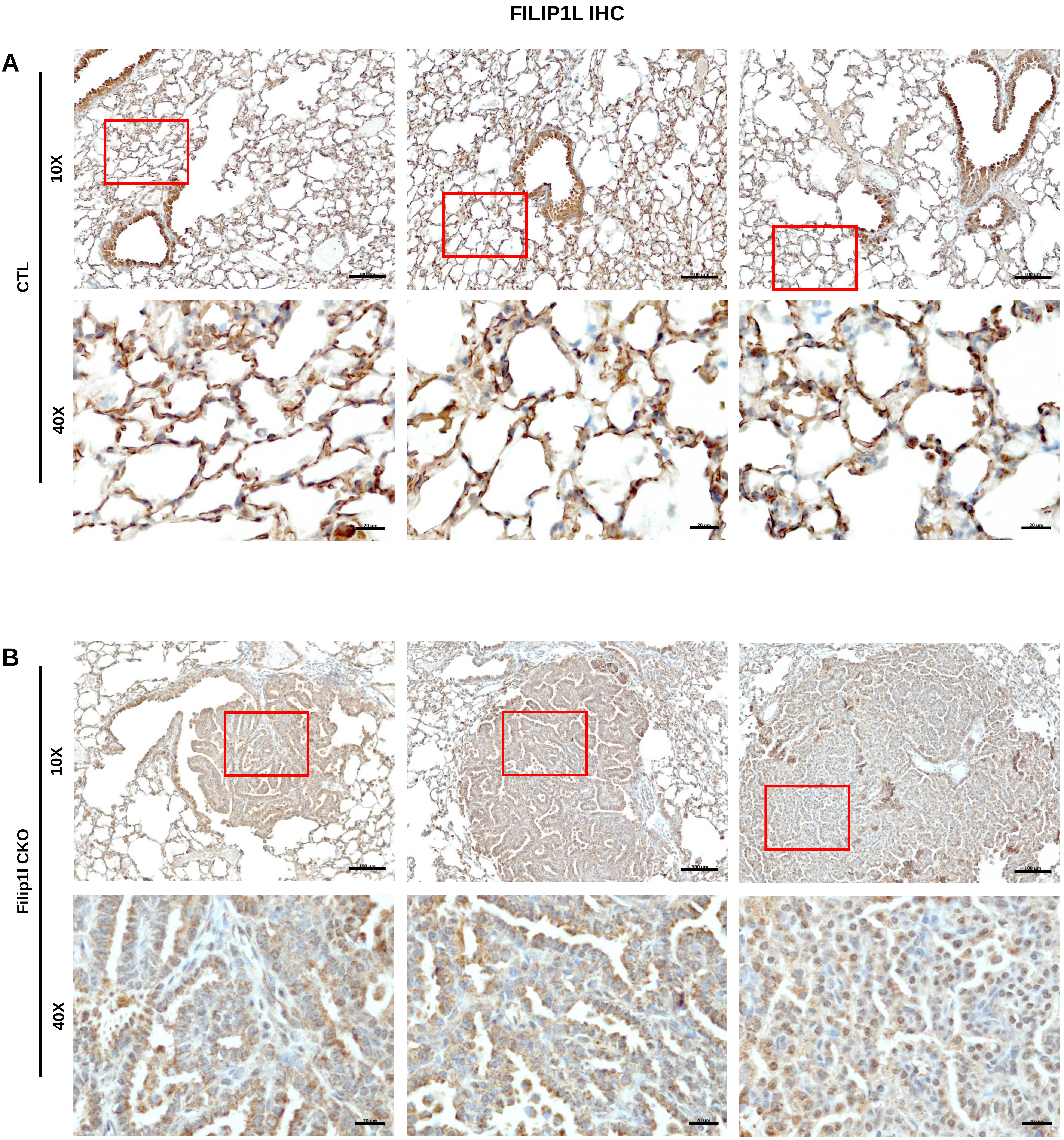

Supplementary Figure S4. Lung tissues from Lenti-Cre-treated C57BL6/J (CTL) **(A)** and Filip1l$^{fl/fl}$ (CKO) **(B)** mice sacrificed at 32 weeks, were fixed and immunohistochemically stained for FILIP1L. Magnified images (40X) from the boxed areas are also shown. Scale bar = 100 µm (10X images); 20 µm (40X images).

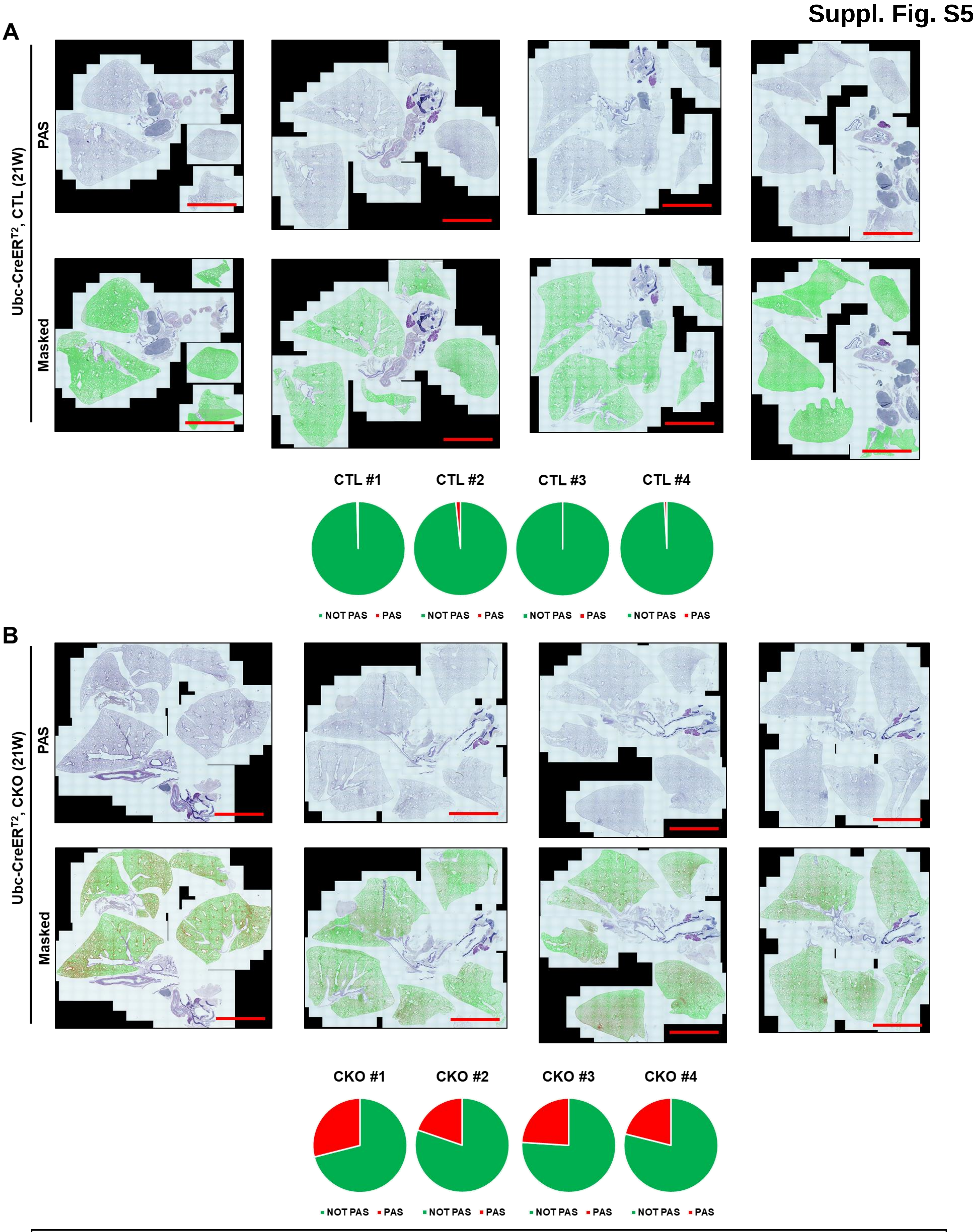

Supplementary Figure S5. Tamoxifen-treated Filip1l$^{fl/fl}$ (CTL) and Filip1l$^{fl/fl}$; Ubc-CreER^{T2} (CKO) mice were sacrificed at 21 weeks, and the lung tissues were subjected to PAS staining. Stitched images of lungs from four representative mice from either CTL **(A)** or Filip1l CKO **(B)** groups are shown. Within each panel, the upper images show the original PAS staining, and the lower images show masked images where PAS-positive and –negative areas are represented by red and green colors, respectively. Quantified PAS-positive areas (out of total lung areas) are also shown in red color in the diagram of bottom side. Scale bar = 5,000 μm.

Suppl. Fig. S6

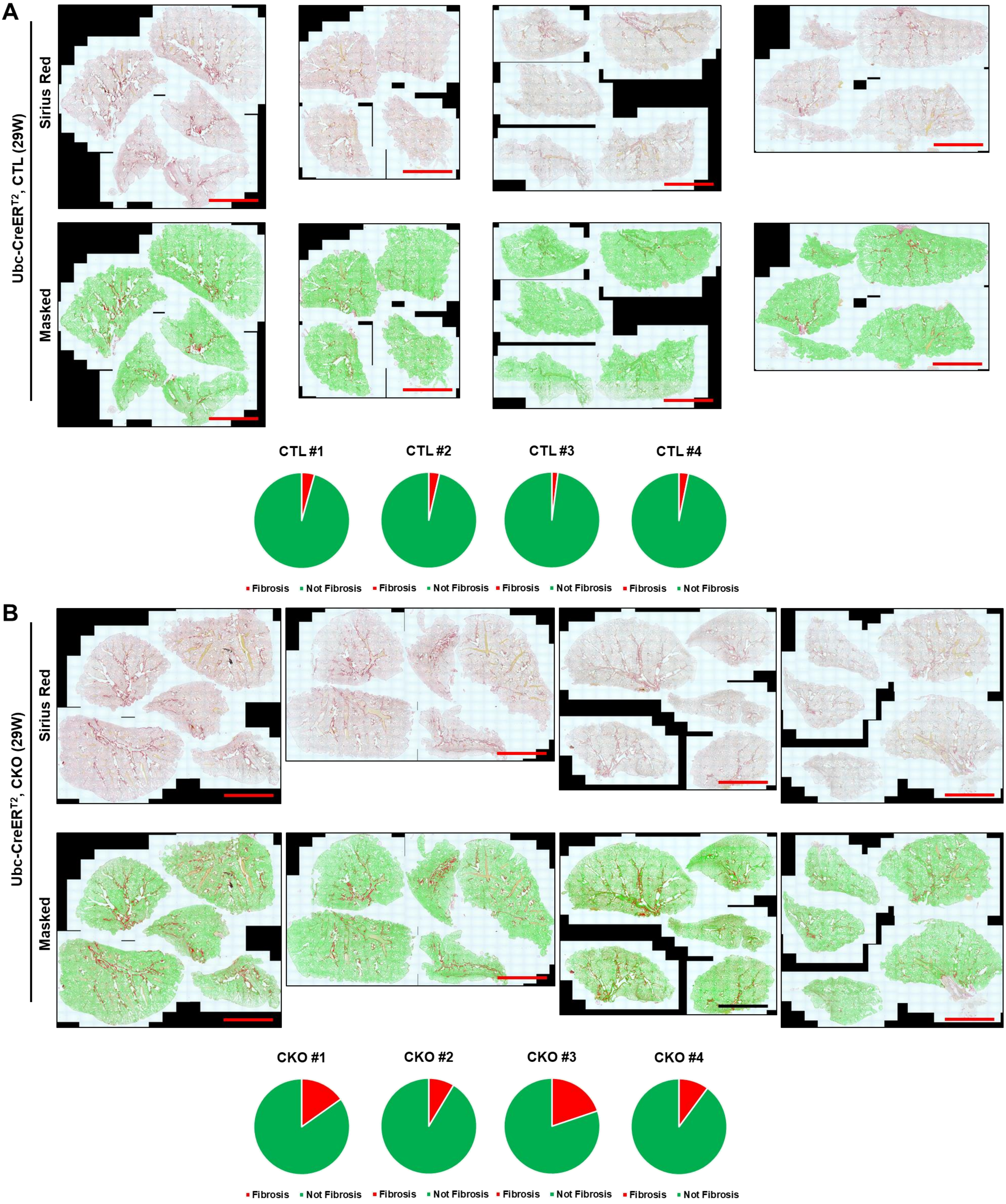

Supplementary Figure S6. Tamoxifen-treated Filip1l$^{fl/fl}$ (CTL) and Filip1l$^{fl/fl}$; Ubc-CreER^{T2} (CKO) mice were sacrificed at 29 weeks, and the lung tissues were subjected to Picro-Sirius Red staining. Stitched images of lungs from four representative mice from either CTL **(A)** or Filip1l CKO **(B)** groups are shown. Within each panel, the upper images show the original Picro-Sirius Red staining, and the lower images show masked images where Picro-Sirius Red-positive and –negative areas are represented by red and green colors, respectively. Quantified Picro-Sirius Red-positive areas (out of total lung areas) are also shown in red color in the diagram of bottom side. Scale bar = 5,000 μm.

Suppl. Fig. S7

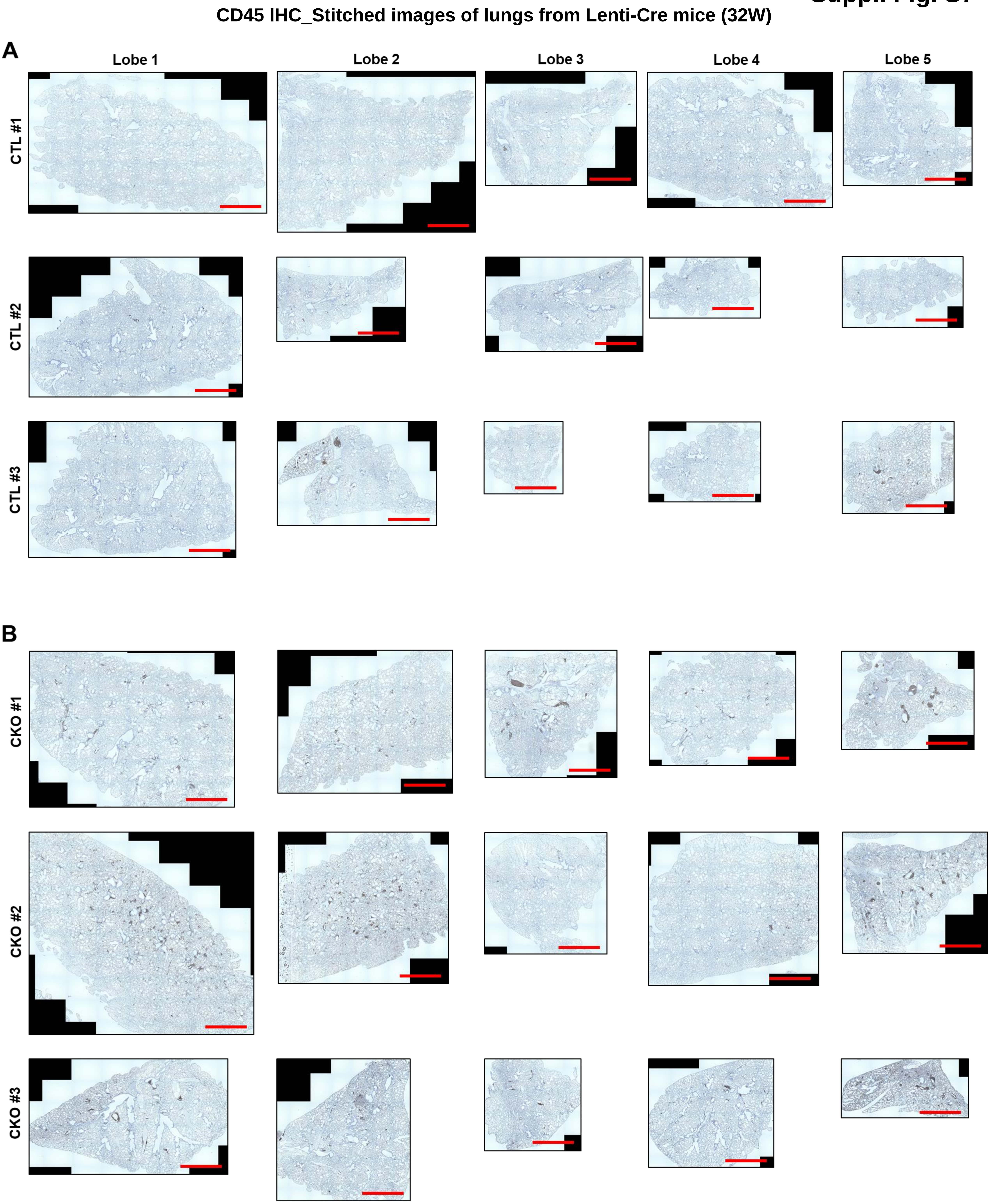

Supplementary Figure S7. Lenti-Cre-treated C57BL6/J (CTL) and Filip1l$^{fl/fl}$ (CKO) mice were sacrificed at 32 weeks, and the lung tissues were immunohistochemically stained for CD45. Stitched images of CD45-stained all the lung lobes from three CTL and three Filip1l CKO mice are shown. Scale bar = 5,000 μm.

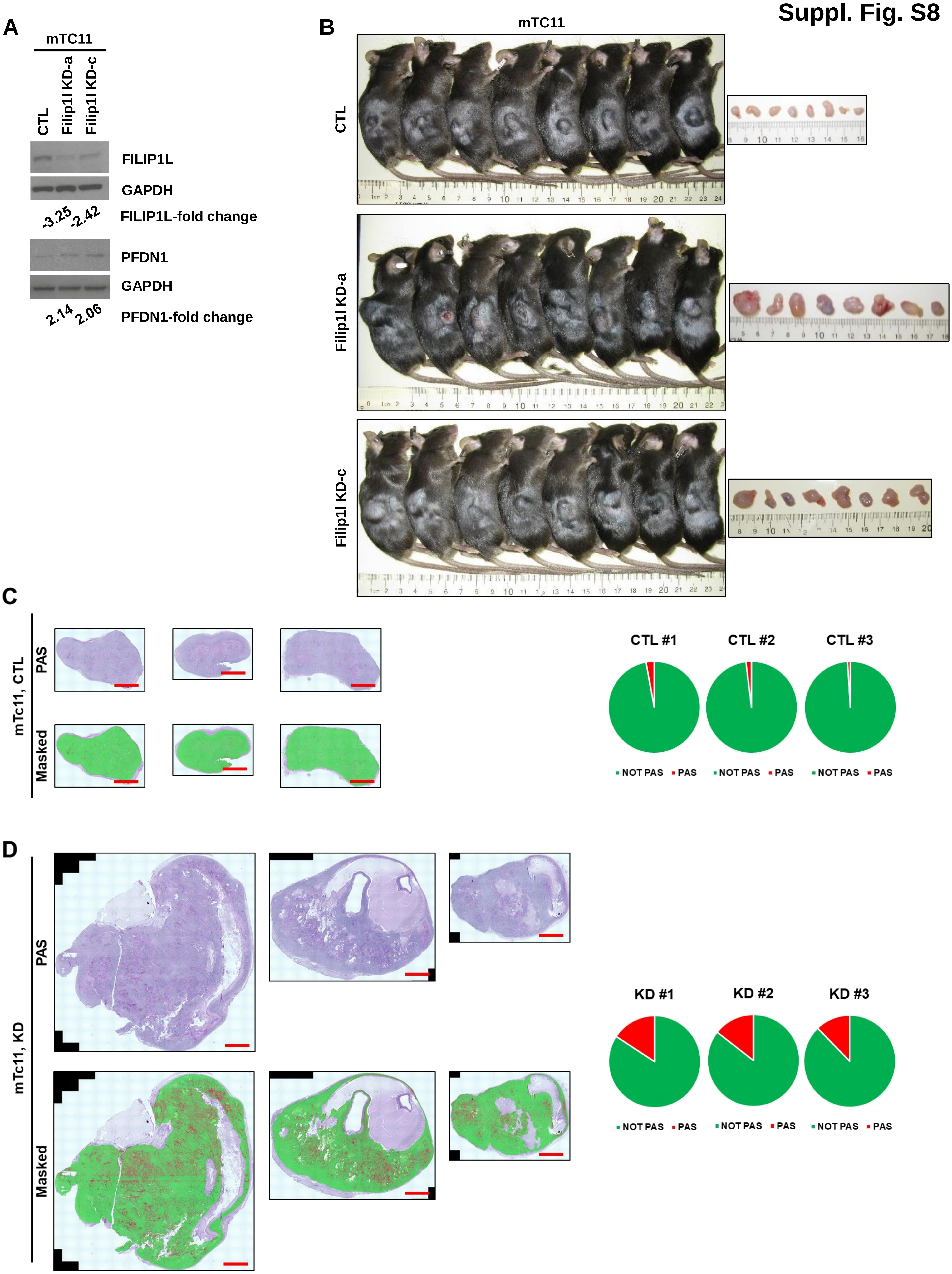
A
mTC11
CTL
Filip1l KD-a
Filip1l KD-c
FILIP1L
GAPDH
-3.25
-2.42
FILIP1L-fold change
PFDN1
GAPDH
2.14
2.06
PFDN1-fold change
B
mTC11
CTL
Filip1l KD-a
Filip1l KD-c
C
mTc11, CTL
PAS
Masked
CTL #1
CTL #2
CTL #3
NOT PAS
PAS
D
mTc11, KD
PAS
Masked
KD #1
KD #2
KD #3
NOT PAS
PAS

Supplementary Figure S8. (A) Filip1l knockdown was achieved by stable expression of Lentiviral shRNA in mTC11 mouse lung cancer cells. Two individual shRNA for Filip1l (constructs a and c) were used. Control clones were made with scrambled shRNA. FILIP1L, PFDN1 and GAPDH control were detected by immunoblotting. By densitometric quantification, the ratio of FILIP1L/GAPDH and PFDN1/GAPDH was determined. Fold changes of either FILIP1L or PFDN1 in Filip1l-knockdown (KD) clones over control (CTL) clones were calculated by dividing the ratio of either FILIP1L/GAPDH or PFDN1/GAPDH (KD/CTL). **(B-D)** mTC11 clones of either control (CTL) or Filip1l-knockdown (KD) derivatives were subcutaneously injected into the C57BL6/J mice (8 mice per cell line). **(B)** Pictures of mice and xenograft tumors at the time of sacrifice (24 days) are shown. **(C-D)** Stitched images of three representative tumors from either control **(C)** or Filip1l-knockdown **(D)** groups are shown. Within each panel, the upper images show the original PAS staining, and the lower images show masked images where PAS-positive and –negative areas are represented by red and green colors, respectively. Quantified PAS-positive areas (out of total tumor areas) are also shown in red color in the diagram of right side. Scale bar = 2,000 μm.

Suppl. Fig. S9

A

Group 1: Basic Immunophenotyping (Lymphocytes, T cells, CD8+ T cells, CD4+ T cells, B cells and NK cells)

Immunogen	Fluorescent Marker	Manufacturer	Catalog#
Viability	eFluor 506	Invitrogen	65086614
CD45	BUV 563	BD Biosciences	612924
CD3e	AF 700	Biolegend	152316
CD4	BUV 737	BD Biosciences	612761
CD8a	BV 570	Biolegend	100740
CD19	BUV 395	BD Biosciences	563557
NKp46	PE-Vio 770	Miltenyi	130112203

Group 2: T cell Function (Naive, Effector, Memory, Extravasation, Activation and Exhaustion Markers)

Immunogen	Fluorescent Marker	Manufacturer	Catalog#
CD44	BV 650	Biolegend	103049
CD62L	APC/Fire 750	Biolegend	104450
PD1	FITC	Biolegend	135214
TIM3	BV 605	Biolegend	119721
LAG3	BV 785	Biolegend	125219

Group 3: Myeloid Cell Immunophenotyping (Dendritic Cells, Macrophages, Monocytes and Neutrophils)

Immunogen	Fluorescent Marker	Manufacturer	Catalog#
Ly6G	BV 711	Biolegend	127643
Ly6C	PE/Dazzle 594	Biolegend	128044
CD11c	VioBlue	Miltenyi	130110706
CD11b	BUV 661	BD Biosciences	612977
F4/80	PerCP-Vio 700	Miltenyi	130118327
CD80	BV 480	BD Biosciences	746775
CD206	APC	Biolegend	141708
MHC II	PE/Cy5	Biolegend	107612

Group 4: Intracellular Markers (Tregs and Proliferating Cells)

Immunogen	Fluorescent Marker	Manufacturer	Catalog#
FoxP3	AF 647	Biolegend	320014
Ki67	PE	Miltenyi	130120417

Suppl. Fig. S9

B

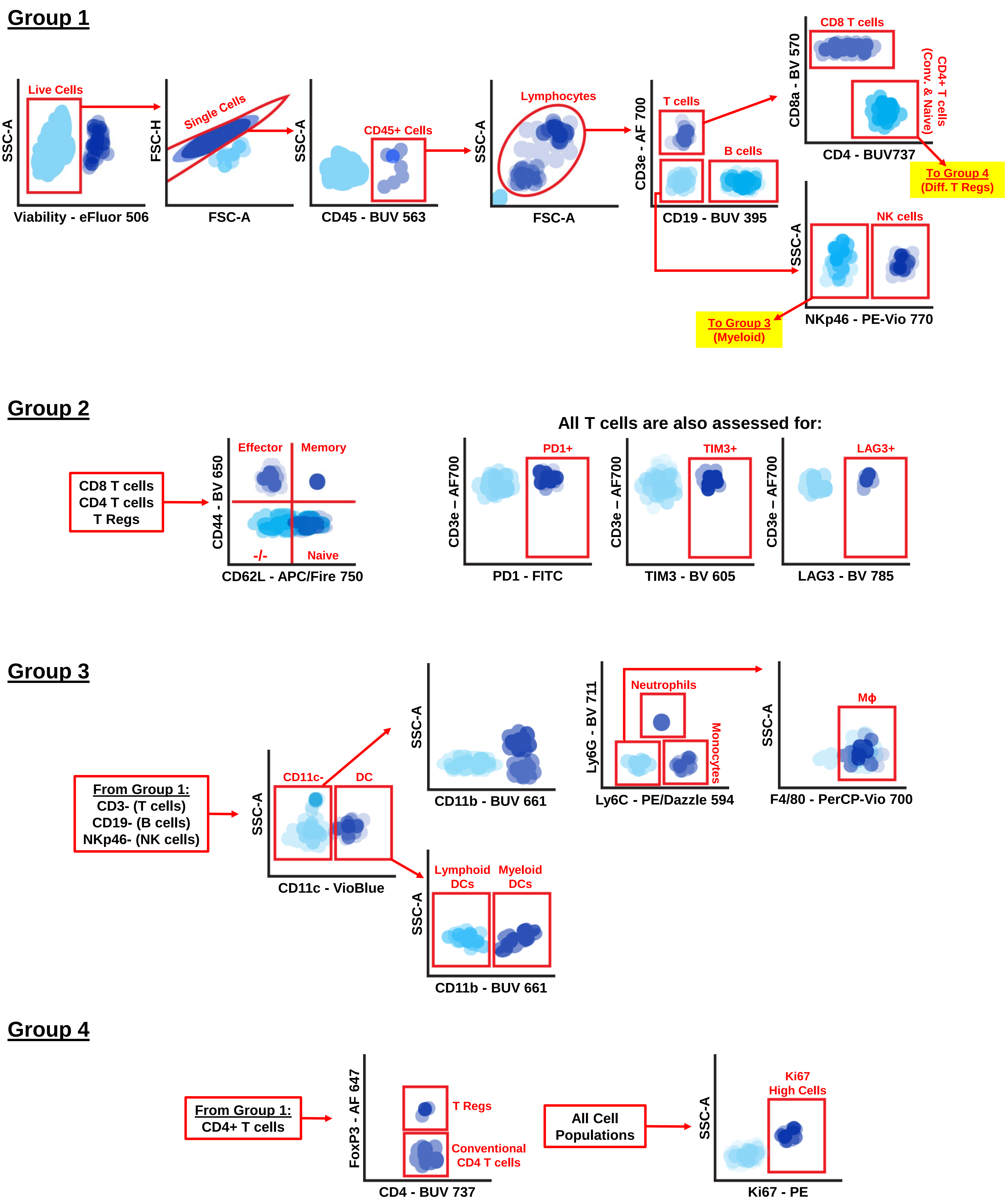

Suppl. Fig. S9

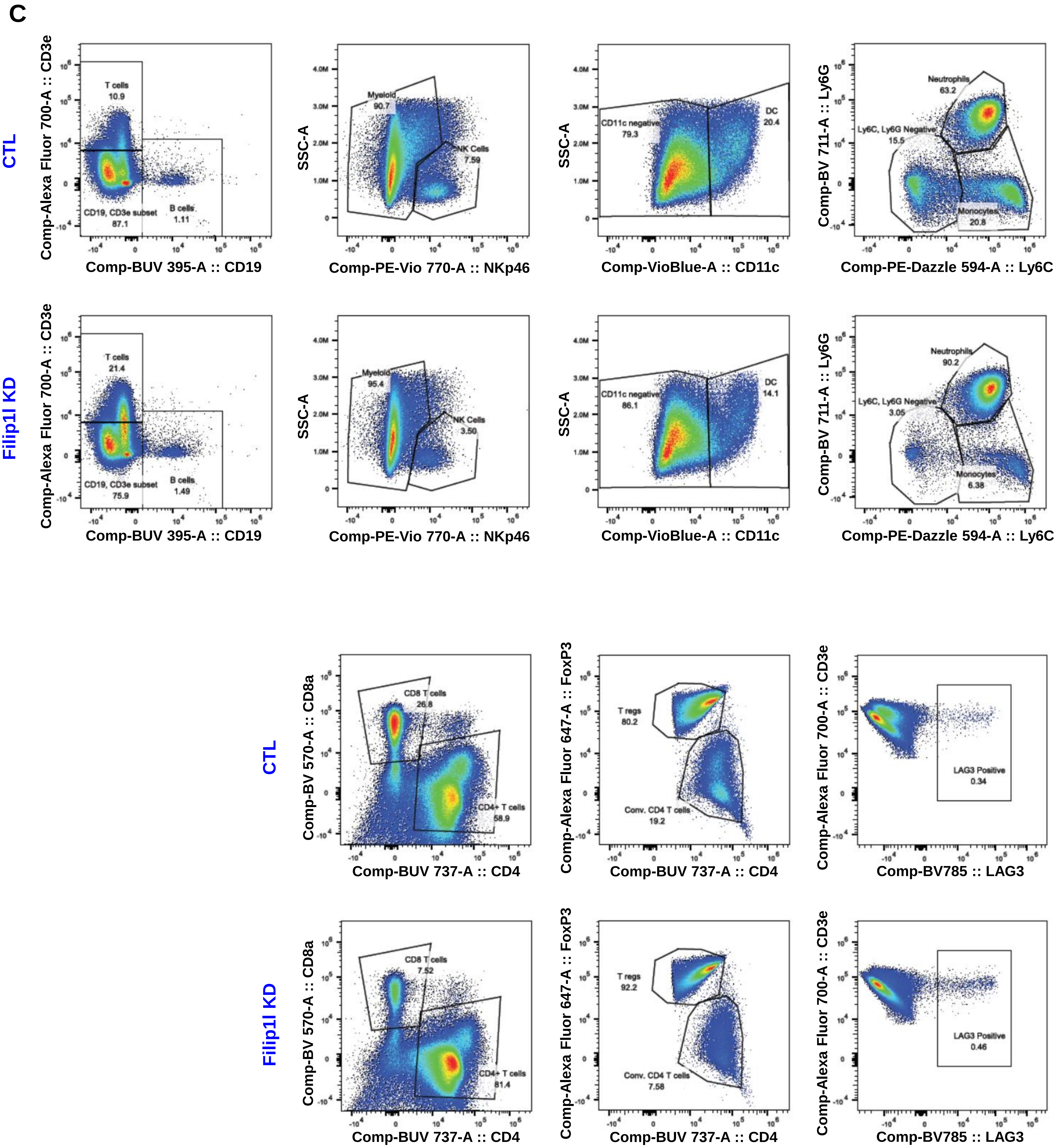

Supplementary Figure S9. Fresh tumors from both control and Filip1l-knockdown groups of mTC11 clones were harvested at day 24 after subcutaneous injection. Dissociated cells were immunofluorescently stained with various markers for immune cells and subjected to FACS analysis. **(A)** The list of the primary antibody conjugated with the secondary antibody is shown in the tables of groups 1-4. **(B)** FACS schemes are shown by flowchart. **(C)** Representative FACS data from the selected FACS schemes are compared between control (CTL) and Filip1l-knockdown (KD) groups.

Suppl. Fig. S10

A

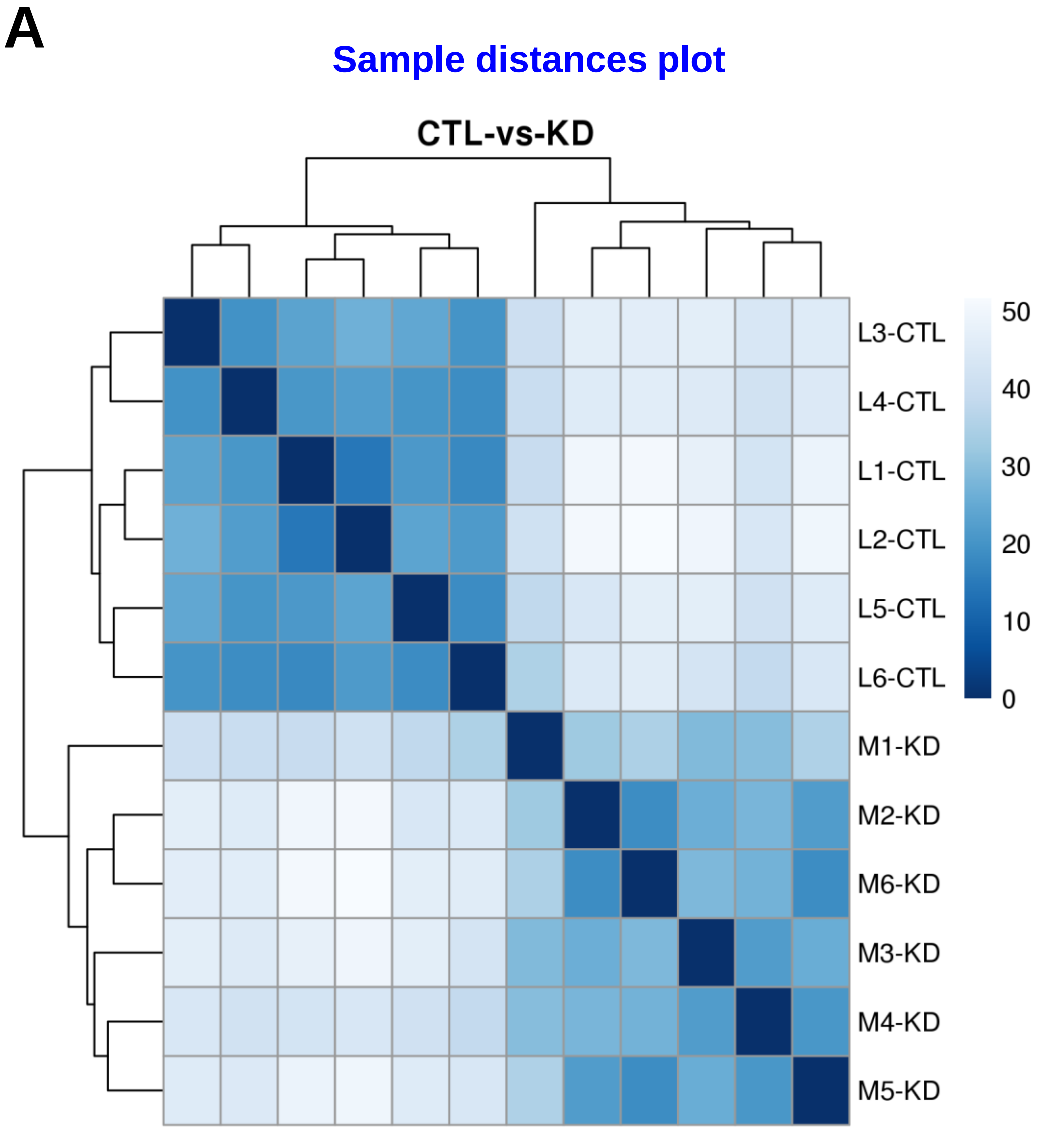

B

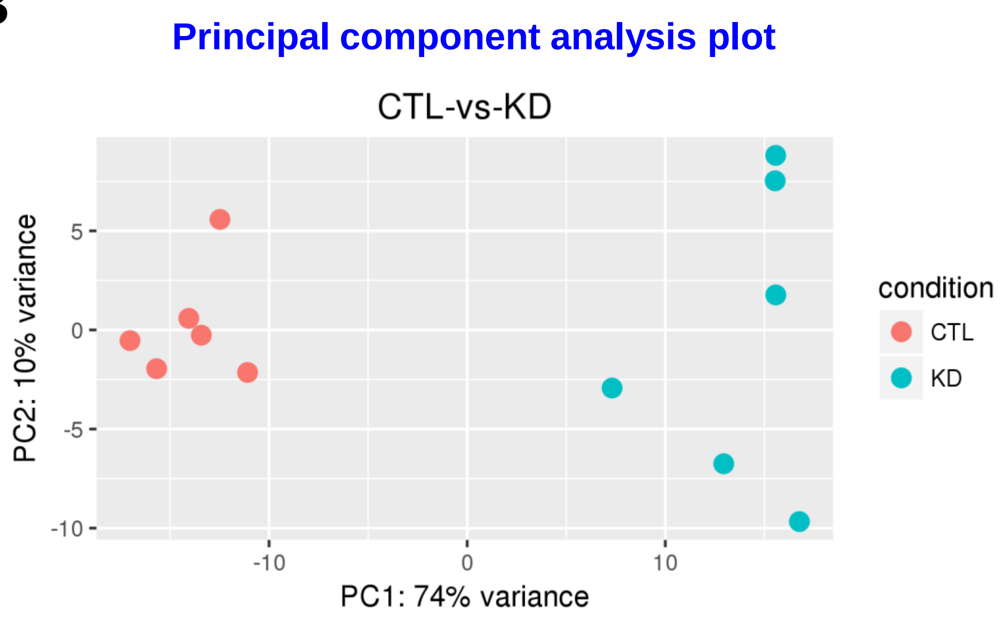

Supplementary Figure S10. RNA-Seq analysis was performed on the frozen mTC11-syngeneic allograft tumors between control (CTL) and Filip1l-knockdown (KD) groups (as described in **Figure 6A-D**; 6 tumors each). **(A)** Sample distances plot. The overall similarity among samples were assessed by the euclidean distance between samples. This method was used to examine which samples are similar/different to each other and if they fit to the expectation from the experiment design. The shorter the distance, the more closely related the samples are. Samples were then clustered by using the distance. **(B)** Principal component analysis plot. A principal component analysis is another way to reveal the similarity between samples based on the distance matrix. Samples were projected to a 2D plane spanned by their first two principal components. This type of plot is useful for visualizing the overall effect of experimental covariates and batch effects. The x-axis is the direction that explains the most variance and the y-axis is the second most. The percentage of the total variance per direction is shown in the label.

SUPPLEMENTARY INFORMATION

Target protein for antibody	Vendor	Catalogue#	Application
FILIP1L	Aviva	ARP52360_P050	IHC
FILIP1L	Atlas	HPA043133	IB
PFDN1	Thermo	PA5-52304	IB
PFDN1	Abcam	ab151708	IHC
GAPDH	Sigma	MAB374	IB
Ki-67	Abcam	ab16667	IHC, IF
TTF1	Abcam	ab76013	IHC
p63	Cell Signaling	39692	IHC
CD45	Cell Signaling	70257	IHC
Ly6G	Cell Signaling	87048	IHC
FoxP3	Cell Signaling	12653	IHC
Non-phospho (Active) β-Catenin (Ser33/37/Thr41)	Cell Signaling	8814	IHC, IF
Alcian Blue Stain 1%, pH 2.5	Newcomer Supply	1003B	
Periodic Acid Schiff (PAS) Stain Kit	Newcomer Supply	9162B	
Trichrome Stain Kit	Abcam	ab150686	
Picro Sirius Red Stain Kit	Abcam	ab150681	
**IB: Immunoblot, IF: Immunofluorescence, IHC: Immunohistochemistry.**			

SYBR Primer list

Mouse Gene (official symbol)	Forward primer	Reverse primer
Filip1l	CAGACTACAAGAGCCTCATTCC	AGCACAGACTCCTCCTCATTA
Actb	GGCTGTATTCCCCTCCATCG	CCAGTTGGTAACAATGCCATGT
Rpl7	CCTTGATTGCTCGGTCTCTT	GAAGCGTTTCCCGACTGTAT
Ly6g	GCGTTGCTCTGGAGATAGAA	GTCTTCACGTTGACAGCATTAC
Foxp3	TCGGGTACACCCAGGAAA	TCACAACCAGGCCACTTG
Agr2	CCAAAGACACCACAGTCAAATC	GCTTCTTCGTATGTCTGAGTCC
Nlrp6	GAGGTCGGATATTCGAGATGTG	GCTCGCGGTACTTCTTCTT
Muc5ac	ATGAAGCTTGATGGCCTAGTT	TTCAGGTAGCCATTGCTGAG
Muc5b	GAAGAGCAGTGGCTATGTGAA	CCACACGTCTGGTTGATGTAT
Muc3	GTGCACCAGCCTCTTCTATG	GGTTACTGTCACACTCACTTCC
Muc4	CCTGTATTCTTGCCTCCTCTTG	TGTGAGCTCATTTGGGATGTT
Muc13	GTCTCTGTCGTCATGTGTGAA	CTCTTGATAGCCCACAGAGTTT
Muc20	CGACGCATCCTACAACATCT	GAGGAAGCCTCCATCTTCAC
Gkn1	GGAATAGCCTCTGGGACTATGA	GCATGGCATCCTTGTTCATTC
Nox1	CTCTCCAGCCTATCTCATCCT	AGGCCAGCAATACTGGTAAAT
Dpp4	CATGGTCACCAGAAGGTCATAA	CTCCTGTCGATGTGATCCTATG
Pten	CCGCCAAATTTAACTGCAGAG	AGATCTTCACAGAAGGGTTTGAT
Hhip	ACATTCTTCGGGTTGTGGAATA	CCAAGATGCTTTCGGTGGA
Ndnf	CCAAACTTTCTTCTTCCCACTAGA	GGTCCTTGAAGTGAGTGGTAAC
Il1a	CCGTGTTGCTGAAGGAGTT	GCTGATGTGAAGTAGTTCTTAGAGT
Il1b	Qiagen, Mm_Il1b_2_SG QuantiTect Primer Assay, QT01048355	
Il6	GAAATGATGGATGCTACCAAACTG	ACTCCAGGTAGCTATGGTACTC
Tnf	GCTCTTCTGTCTACTGAACTTCG	AGGGTCTGGGCCATAGAA
Nos2	GGTCTTTGAAATCCCTCCTGAT	AGTGCATACCACTTCAACCC
Cxcl5	ATCGCTAATTTGGAGGTGATCC	AGACAGACCTCCTTCTGGTT
Mep1b	GGACTTCAGTGACTATGACCTTC	ACTTTGGATCATGCCACAGATA
Reg3b	CTCCTACTGCTATGCCTTGTTT	CTATTGAGCACAGATACGAGGTG
Fut9	TCTCTGCTTTCCATGCTATGTT	CCAGGATGATGCAGACGATTAG
Bpifb1	GCCTTCAACTTATGACCAGGAG	GTGCTCATTTGGAACTCCACTA
Il10	TTGAATTCCCTGGGTGAGAAG	TCCACTGCCTTGCTCTTATTT
Mmp7	GAGATGCTCACTTTGACAAGGA	CCAGAGAGTGGCCAAATTCA
Vegfa	ACGTCAGAGAGCAACATCAC	GTGCTGTAGGAAGCTCATCTC
L1cam	GTACCCGGACCATCATTCAAA	GCTGGAGTTTGTGGGAAAGA
Egfr	GGTCCTTGGGAACTTGGAAAT	GTTGAGGGCAATGAGGACATAG
Ret	CAATGAGACTACTGGCCTTCTC	CAGAAAGACCTGGAGGAAGATG
Dll1	CATACAGACTCTCCCGATGAC	AGACCATTCTTCTCCCACAG

Quantification procedures for Periodic Acid Schiff (PAS) and Picro-Sirius Red staining:

[**PAS**]/[**Sirius Red**] stitched images were acquired by EVOS FL Auto microscope (Thermo Fisher Scientific). The total positive [**PAS**]/[**Sirius Red**] areas were selected and quantified using the Celleste software (Thermo Fisher Scientific, Version 4.1.1).

Procedure:

1) Regions of Interest (ROI) are selected using the Multiple ROIs feature to solely quantify the sample, and avoid areas of unnecessary background. The ROI selection depends on the staining pattern:

    a. Using the Smart Segmentation Feature, Positive, Negative, and Background classes were created, and small representative areas are manually selected for each class. The program then automatically indicates the areas by masking them.

        i. **For PAS Quantification:**

        The Positive and Negative PAS areas were masked by the colors of red and green, respectively. Red is used to mask the dark purple/magenta stain whereas green is used to mask the blue and light pink stains. Background areas were shown as any area excluded from the quantification (no masking).

        ii. **For Sirius Red Quantification:**

        The Positive and Negative Sirius Red areas were masked by the colors of red and green, respectively. Red is used to mask the dark red stain whereas green is used to mask the light red and yellow stains. Background areas were shown as any area excluded from the quantification (no masking).

    b. After proper representative areas are selected, the selections are saved for future use on other images in order to make selection more consistent and automatic across images.

2) Once masked, the Count feature is selected, and a sum of the area of the pixels for each class (Positive [**PAS**]/[**Sirius Red**] and Negative [**PAS**]/[**Sirius Red**]) is calculated and quantified. These values are graphed to show the relative percentages of how much area on the image is positive or negative. The masked image is also shown as a visual representation of the quantification.

3) Repeat for all images, display the data on a bar graph and perform a t-test.

__**Quantification procedures for Total-β-Catenin and Cytosolic-β-Catenin:**__

Steps 1-2 were performed in ZEN 3.1 software (Zeiss).

Steps 3-8 and 10-15 were performed in CellProfiler 4.2.1 software.

Steps 9 and 16 were performed in Microsoft Excel software.

1. Acquire Z-stack images of desired tissue (IF-stained for β-Catenin (green) and DAPI (blue)) from randomly selected representative areas.
2. Create a maximum intensity projection-TIFF image from all Z-stacks.

For Total-β-Catenin Quantification:

3. Split the RGB TIFF image into two grayscale images: β-Catenin and DAPI.
4. Apply threshold to the grayscale images (from step 3) to generate thresholded images.
5. Using thresholded images generated in step 4, create an image mask of the β-Catenin image on the DAPI image. This will leave only DAPI images with positive β-Catenin staining.
6. Calculate the sum of pixel intensity in the β-Catenin grayscale image (from step 3).
7. Measure the image area occupied by DAPI using DAPI thresholded image (from step 5).
8. Divide the total pixel intensity (from step 6) by the DAPI area (from step 7) to calculate the average pixel intensity per DAPI area.
9. Repeat for all images, display the data on a box and whisker plot and perform a t-test.

For Cytosolic-β-Catenin Quantification:

10. Perform steps 3-4 of the **Total- β-Catenin quantification**, with increase in the threshold value of the β-Catenin image:
    a. The threshold value of β-Catenin image must be increased to isolate **membrane β-Catenin image.**
11. Remove membrane β-Catenin image (from step 10) from total β-Catenin grayscale image (from step 3) using ImageMath module to leave **cytosolic β-Catenin image.**
12. Using the images created in steps 10 and 11, create an image mask of the cytosolic β-Catenin image on DAPI image. This will leave only DAPI images with positive β-Catenin staining.
13. Calculate the sum of the pixel intensity in the cytosolic β-Catenin image (from step 11).
14. Measure the image area occupied by DAPI using DAPI thresholded image (from step 12).
15. Divide the total pixel intensity (from step 13) by the DAPI area (from step 14) to calculate the average pixel intensity per DAPI area.
16. Repeat for all images, display the data on a box and whisker plot and perform a t-test.
